# Multi-Omics Reveals the Effect of Population Density on the Phenotype, Transcriptome and Metabolome of *Mythimna separata*

**DOI:** 10.3390/insects14010068

**Published:** 2023-01-10

**Authors:** Sibo Wang, Hongjia Yang, Yushuo Hu, Chunyu Zhang, Dong Fan

**Affiliations:** College of Plant Protection, Northeast Agricultural University, Harbin 150030, China

**Keywords:** *Mythimna separata*, phase change, transcriptomic, metabolomic, energy metabolism

## Abstract

**Simple Summary:**

*Mythimna separata* living in the environment with high population density show characteristics of melanization and overeating and cause serious damage to crops. In order to explore the impact of population density on *M. separata*, we studied the gene expression and metabolite accumulation of *M. separata* under different population densities through transcriptome and metabolome analysis. The results showed that the insulin-like signaling pathway influences the phase change of *M. separata*. Compared with solitary *M. separata*, the gregarious type had a faster energy consumption rate and stronger protein digestion and absorption capacity, while the transcription and translation processes were inhibited. This study explains the molecular mechanism of the phase change and the overeating behavior of *M. separata* under high population density.

**Abstract:**

Population-density-dependent polymorphism is important in the biology of some agricultural pests. The oriental armyworm (*Mythimna separata*) is a lepidopteran pest (family Noctuidae). As the population density increases, its body color becomes darker, and the insect eats more and causes greater damage to crops. The molecular mechanisms underlying this phase change are not fully clear. Here, we used transcriptomic and metabolomic methods to study the effect of population density on the differentiation of second-day sixth instar *M. separata* larvae. The transcriptomic analysis identified 1148 differentially expressed genes (DEGs) in gregarious-type (i.e., high-population-density) armyworms compared with solitary-type (low-population-density) armyworms; 481 and 667 genes were up- and downregulated, respectively. The metabolomic analysis identified 137 differentially accumulated metabolites (DAMs), including 59 upregulated and 78 downregulated. The analysis of DEGs and DAMs showed that activation of the insulin-like signaling pathway promotes the melanization of gregarious armyworms and accelerates the decomposition of saccharides, which promotes the gregarious type to take in more food. The gregarious type is more capable of digesting and absorbing proteins and decreases energy consumption by inhibiting transcription and translation processes. The phase change traits of the armyworm are thus attributable to plasticity of its energy metabolism. These data broaden our understanding of the molecular mechanisms of insect-density-dependent polymorphism.

## 1. Introduction

Polyphenism is the phenomenon of two or more different phenotypes arising from the same genotype, which is widespread in insects [1]. Phenotypic variation in some insects, such as locusts, aphids, planthoppers and the larvae of some moths, is influenced by increases or decreases in the population density [2,3,4,5,6]. The phenotype of these insects at low density is known as solitary type and at higher density as gregarious type. High density can not only modify the appearance of these insects, such as the depth of body color or the length of wings, but also lead to behavioral and physiological changes. For example, the body color of the gregarious oriental migratory locust (*Locusta migratoria*) changes from green to brown, whereas that of the gregarious desert locust (*Schistocerca gregaria*) changes from grayish-yellow to brilliant yellow, upon going from low to high population density. Gregarious locusts can migrate far away and have a higher resistance to pathogenic fungi [7,8]. The density-dependent polyphenism of insects is an essential manifestation of insect adaptation to the environment, and it also creates difficulties in the prevention and control of agricultural pests.

Insects can perceive the change in density in simple ways, such as by vision, smell and touch [9,10,11], but the molecular regulatory mechanisms behind the density-dependent polyphenism are complex. The β-carotene-binding protein (βCBP) of gregarious oriental migratory locusts binds the red β-carotene pigment and forms a black pattern on the body surface, together with the blue biliverdin pigment and yellow carotenoids [12]. Carnitine may mediate phase changes in locusts by regulating lipid metabolism and affecting the nervous system [13]. The two insulin-like receptors—InR1 and InR2—play opposite roles in controlling wing length by regulating the transcriptional activity of the forkhead transcription factor (FOXO) in the brown planthopper (*Nilaparvata lugens*, BPH) [14]. [His7]-corazonin and the juvenile hormone play an important role in the pigment deposition of migratory locusts [15]. The juvenile hormone has also been proven to affect the wing type differentiation of aphids [16].

*Mythimna separata* (Lepidoptera, Noctuidae), which is usually called the oriental armyworm, is a common pest of food crops in some Asian countries and has a long history of infestation in China. This insect has the habit of voracity and long-distance migration, meaning that it causes serious crop damage and is difficult to prevent [17]. The larvae of *M. separata* show typical density-dependent polyphenism. In the case of feeding on corn leaves, solitary larvae are green; their body color gradually darkens with the increase in density, and finally becomes black. Compared with solitary larvae, gregarious larvae are more active and voracious. They develop faster, are more tolerant to hunger and are better able to adapt to unpalatable food [18]. The hemolymph of fifth instar gregarious-type larvae has higher phenol oxidase activity, total blood cell count, octopamine content and antimicrobial activity against multiple pathogens compared with fifth instar solitary-type larvae [19,20].

The study of insect density-dependent polyphenism can deepen our understanding of the biology of agricultural pests and help us to formulate more scientific control strategies. In this study, we measured and analyzed the transcriptome and metabolome of gregarious and solitary *M. separata* and identified differentially expressed genes and differentially accumulated metabolites in gregarious compared with solitary armyworms. The purpose of this study was to define the biochemical and molecular changes in the phase change process of *M. separata*, so as to further understand the mechanisms.

## 2. Materials and Methods

### 2.1. Insect Materials and Treatments

*M. separata* used in this experiment were collected from the Xiangyang Experimental Demonstration Base of the Northeast Agricultural University, China, and continuously cultivated for several generations in the laboratory. Larvae were transferred into plastic boxes within 10 h of hatching. Gregarious armyworms were reared in plastic boxes (0.3 L) with 10 larvae per box in an artificial climate incubator. Solitary armyworms were raised in another incubator, with each larva in a plastic box (0.3 L). All larvae were cultured at relative humidity 65 ± 5% and 25 ± 1 °C, with a photoperiod of 14 h light and 10 h dark. All larvae were fed fresh, clean corn leaves. The food was always sufficient and fresh throughout the feeding process. The plastic boxes were cleaned every day. The number of gregarious and solitary armyworms used in the entire study exceeded 100.

### 2.2. Growth Record and Sampling

After larvae grew to the fourth instar, the head, back and side of the armyworms were photographed and recorded every day. On the second day of the sixth instar, healthy armyworms of uniform size were collected for transcriptomic and metabolomic analyses, including nine gregarious larvae and nine solitary larvae. After collection, the samples were rapidly frozen in liquid nitrogen and stored at −80 °C. Each sample contained one complete larva. To verify the reversibility of the phase change of armyworms, we put 10 fifth instar solitary larvae into a box together (0.3 L) and isolated some fourth instar gregarious larvae. Their changes in appearance were recorded over time.

### 2.3. Transcriptomic Analysis

Three biological replicates were used in this part of the experiment, and three gregarious larvae and three solitary larvae were used. The cDNA library was sequenced by Shanghai Applied Protein Technology Co., Ltd. (Shanghai, China). After a whole larva was crushed and homogenized, total RNA was extracted with Trizol (Invitrogen, CA, USA)). RNA integrity and DNA contamination were analyzed by agarose gel electrophoresis. A NanoPhotometer spectrophotometer was used to detect RNA purity (OD260/280 and OD260/230). An Agilent 2100 Bioanalyzer was used to detect RNA integrity. After samples passed the tests, a Truseq™ RNA Sample Prep Kit for Illumina^®^ (NEB, Ipswich, MA, USA) was used to build the library. After construction of the library, preliminary quantification was performed using a Qubit2.0 Fluorometer, and the library was diluted to 1.5 ng/μL. The Agilent 2100 Bioanalyzer (Agilent Technologies, Santa Clara, CA, USA) was used to detect the adapter size of the library. After confirming that the library size met expectations, Real-Time Quantitative PCR was used to accurately quantify the effective concentration of the library and ensure the library quality. The effective concentration of the library should be >2 nM. After a library was determined to be qualified according to the effective concentration and target offline data volume requirements, libraries with different concentrations were pooled. Illumina sequencing was performed using the HiSeq 2000 Truseq SBS Kit v3-HS (Illumina).

After sequencing was complete, the original sequencing data were filtered for quality and high-quality (clean) data were obtained. Trinity (v2.5.1, Broad Institute, Cambridge, MA, USA) was used for splicing clean reads. The NR, SwissProt, STRING, GO, KEGG and Pfam databases were used for gene function annotation. In RNA-Seq analysis, Salmon was used to evaluate the expression amounts of unigenes and transcripts, and the expression amount was standardized by Transcripts Per Million (TPM). Fragment Per Kilobase of exon model per Million mapped fragments (FPKM) was used to measure relative gene expression. DES eq2v1.22.1 was used for gene differential expression analysis. P_adj_ < 0.05 was used as the criterion for significant difference.

Real-Time Relative Quantitative PCR (qRT-PCR) was used to verify the accuracy of RNA-Seq. Seven DEGs were randomly selected. β-Actin was selected as the internal reference gene. All primer sequences are listed in Table S1. The fluorescent dye SYBR Primer Script RT-PCR Kit Mix (TOYOBO, Osaka, Japan) was used to perform qRT-PCR on a Bio-Rad fluorescent quantitative PCR instrument. The relative expression of genes was measured using the 2^−ΔΔ Ct^ method.

### 2.4. Metabolomic Analysis

Six biological repetitions were used for this part, and six gregarious larvae and six solitary larvae were used. Metabolomic analysis was performed by Shanghai Applied Protein Technology Co., Ltd. The instrument used for analysis was a UHPLC (1290 Infinity LC, Agilent Technologies, Santa Clara, CA, USA) coupled to a quadrupole time-of-flight mass spectrometer (AB Sciex TripleTOF 6600, Sciex, Redwood City, CA, USA). After crushing and homogenizing a whole larva, 800 μL of a mixture of methanol and acetonitrile (1:1 *v*:*v*) was added to the homogenized solution for metabolite extraction for use in subsequent experiments. The mixture was centrifuged at 14,000× *g* at 4 °C for 20 min, and then the supernatant was extracted. A vacuum centrifuge was used to dry the sample. Before LC-MS experiments, a 100 μL mixture of acetonitrile and water (1:1 *v*:*v*) was prepared, and the sample to be tested was completely dissolved in this solvent. The mixture was centrifuged at 14,000× *g* at 4 °C for 15 min, and then the supernatant was extracted for analysis.

For hydrophilic interaction chromatography (HILIC) separation, a 2.1 mm × 100 mm ACQUITY UPLC BEH Amide 1.7 µm column (Waters, Wexford, Ireland) was used. The mobile phase contained solvents A (25 mM ammonium acetate and 25 mM ammonium hydroxide aqueous solution) and B (acetonitrile). The separation process was 95% B for 0.5 min, then linearly reduced to 65% B over 6.5 min, reduced to 40% B over 1 min, maintained at 40% B for 1 min, and then increased to 95% B in 0.1 min. The re-equilibration period employed in this process was 3 min.

The metabolome analysis was carried out in positive- and negative-ion mode. In MS, the Electron Spray Ionization (ESI) source conditions were set to the following: scanning modes positive and negative; ion source gas 1 and gas 2, 60 psi; and curtain gas, 30 psi. Ion spray voltage floating was 5500 V in positive-ion mode and −5500 V in negative-ion mode. The ion source temperature was 600 °C. The detection range of the primary mass-to-charge ratio was 60–1000 Da and the secondary sub ion mass-to-charge ratio was 25–1000 Da. The scanning cumulative time of the primary mass spectrometry was 0.20 s/spectrum and of the secondary mass spectrometry was 0.05 s/spectrum. The secondary mass spectrometry was obtained in Data-Dependent Acquisition (DDA) mode, and the peak intensity screening mode was used. The declustering voltage was 60 V in positive mode and −60 V in negative mode. The collision energy was 35 ± 15 eV. The DDA was set as follows: the range of dynamically excluded isotope ions was 4 Da, and 10 fragment maps were collected for each scan.

After obtaining the mass raw data, ProteoWizard MSConvert was used to convert them to MzXML files. Then, the data were imported into XCMS software. The local database was used to identify metabolites. After processing, the data were uploaded into SIMCA 14.1 software (MKS Data Analytics Solutions, Umea, Sweden) for multivariate analyses. PCA and OPLS-DA were used to extract the main feature components of the mass data. Sevenfold cross-validation and response permutation testing (*n* = 200) were used to assess the robustness of the model. Student’s *t*-test (*p* < 0.05) and first principal component of variable importance in projection values (VIP >1) were used to screen DAMs. The KEGG database was used to search and analyze metabolic pathways.

## 3. Results

### 3.1. Effect of High Density on Phase Change of M. separata

**Figure 1:**
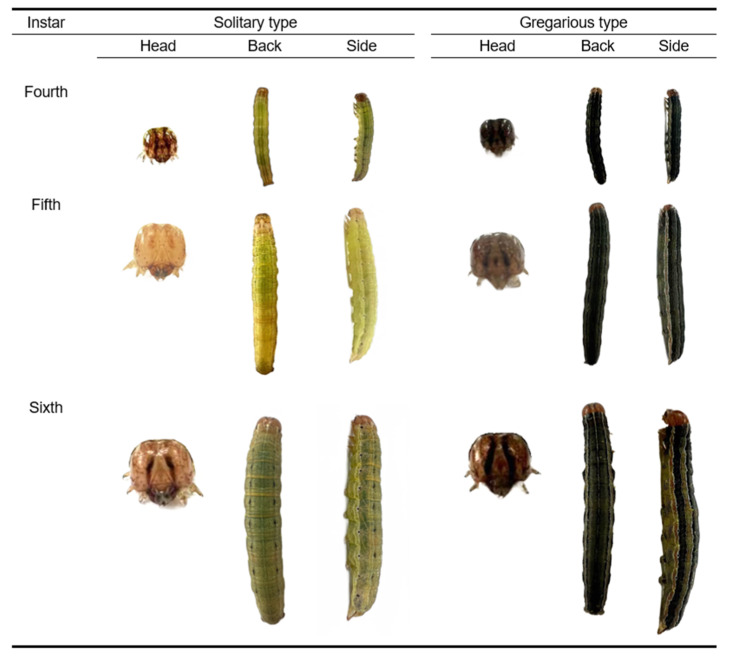
Phenotypes of gregarious and solitary armyworms at different instars.

**Figure 2:**
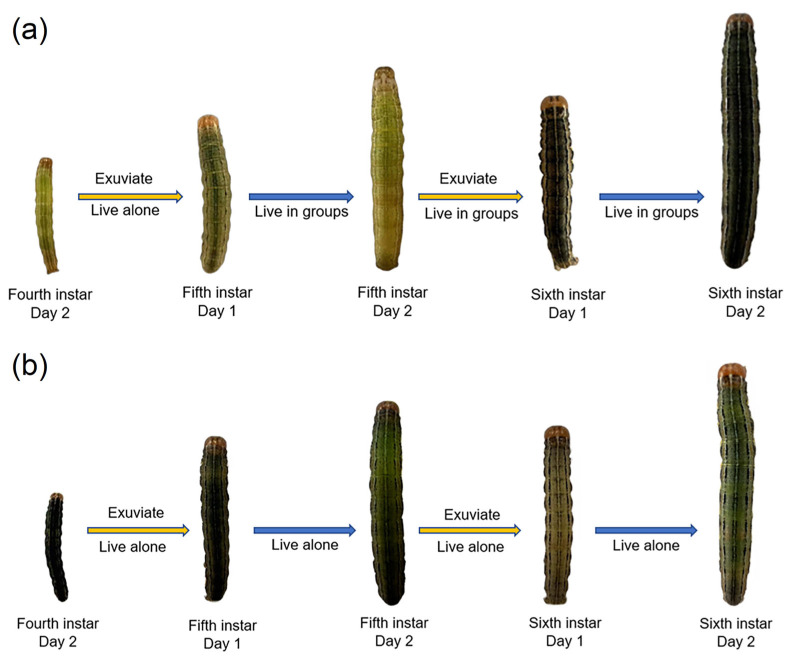
Effect of population density on phase change of armyworms. (**a**) Phenotypic changes of fourth instar solitary armyworms with time after entering a high population density environment. (**b**) Phenotypic changes of gregarious armyworms with time after isolation.

Significant phenotypic differences were found between gregarious and solitary *M. separata* larvae. The body color of gregarious larvae growing to the second day of the sixth instar was black, and the body color of the solitary larvae was green. There was an obvious splayed black pattern in the depression of the head of the gregarious larvae. Fifth instar solitary larvae had only a depression but no pattern in the corresponding position (Figure 1). The body color of fifth instar solitary larvae became darker after entering the high-density environment; after this change, no significant difference was observed between the body color and that of gregarious-type sixth instar larvae (Figure 2a).

We also assessed whether the phase change was reversible. After molting, the body color of isolated fifth instar gregarious larvae was slightly lighter than that of fourth instar gregarious larvae. When these larvae developed to the second day of the sixth instar, their appearance showed marked characteristics of solitary larvae (Figure 2b).

These phenomena demonstrated that the phase change of armyworms is dependent on population density and is reversible.

### 3.2. Effect of Density on Transcriptome of M. separata

**Figure 3:**
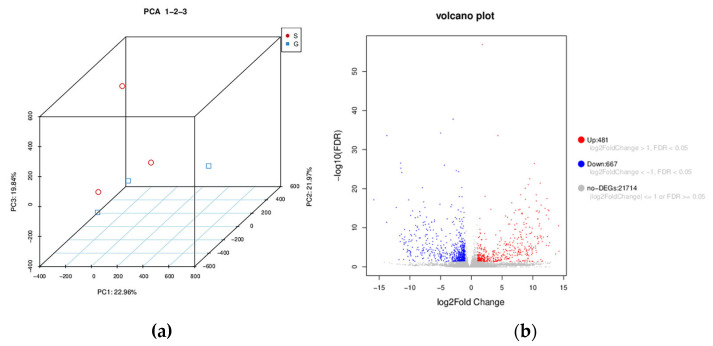
(**a**) Principal component analysis showing statistical clustering of gene expression in gregarious and solitary second-day sixth instar oriental armyworm (*Mythimna separata*) larvae. Red indicates solitary type and blue gregarious type. (**b**) Volcano plot showing the effect of high population density on gene expression in armyworms.

In this part of the experiment, we measured the transcriptome of second-day sixth instar gregarious and solitary *M. separata* larvae. Trinity software assembled 96,195 unigenes from sequencing data after quality control. A list of all unigenes is shown in Table S2. Principal component analysis (PCA) was used to compare gregarious and solitary second-day sixth instar *M. separata*. The score chart showed that gregarious and solitary armyworms were well separated (Figure 3a). Many (1148) differentially expressed genes (DEGs) were identified; 481 upregulated and 667 downregulated genes were discovered in gregarious armyworms compared with the solitary type (Figure 3b). Among them, 192 genes were specifically expressed in the gregarious type, and 136 genes were only expressed in the solitary type. The qPCR validation results are listed in Figure S1.

**Figure 4:**
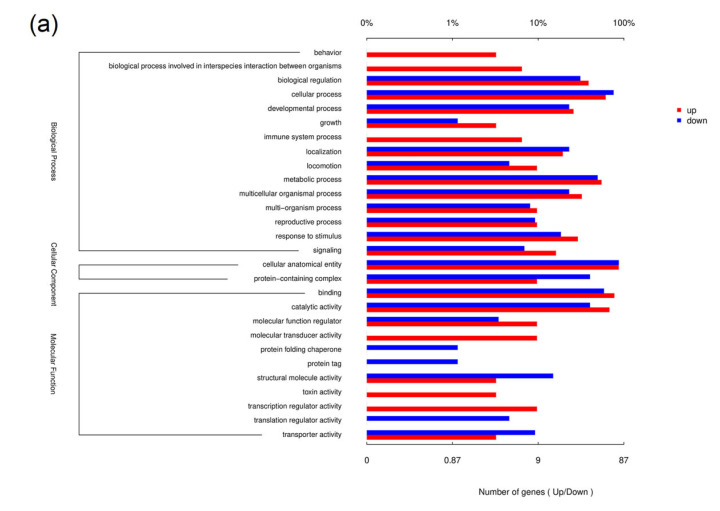

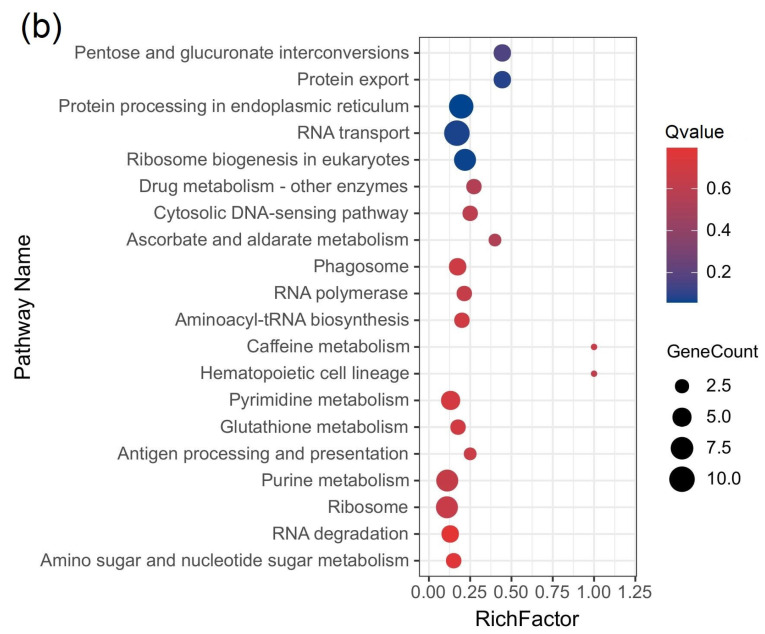
Annotation and enrichment analysis of differentially expressed genes (DEGs) in gregarious armyworms compared with solitary armyworms. (**a**) Column chart of Gene Ontology functional annotation; (**b**) Kyoto Encyclopedia of Genes and Genomes (KEGG) enrichment bubble chart.

**Figure 5:**
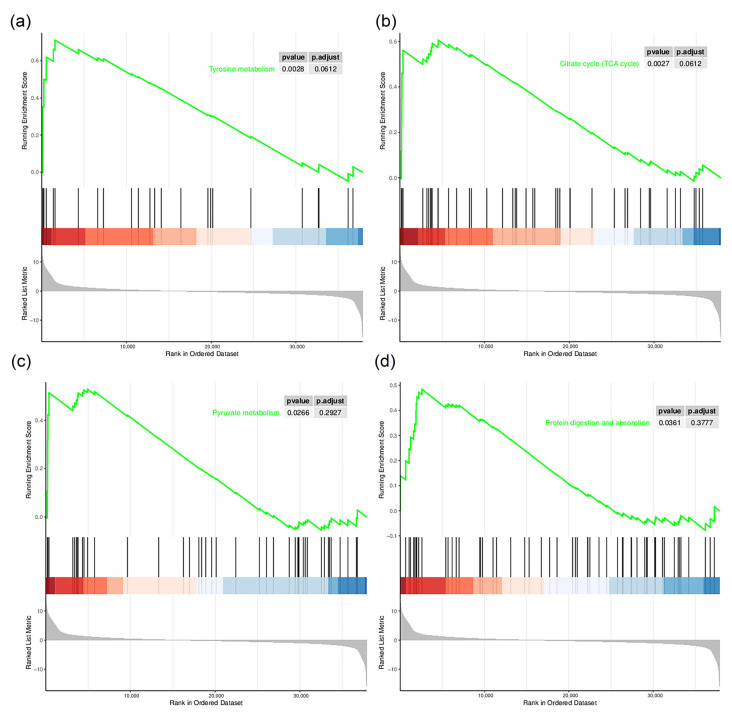
Gene Set Enrichment Analysis (GSEA) of upregulated pathways in gregarious armyworms compared with solitary armyworms. The green curve is an enrichment score line graph, and the score at the peak is the ES value of the gene set. Black lines mark where members of the gene set appear in the gene sequencing list. The grey part is the distribution map of rank values of all genes. (**a**) Tyrosine metabolism; (**b**) the tricarboxylic acid cycle; (**c**) pyruvate metabolism; (**d**) protein digestion and absorption.

**Figure 6:**
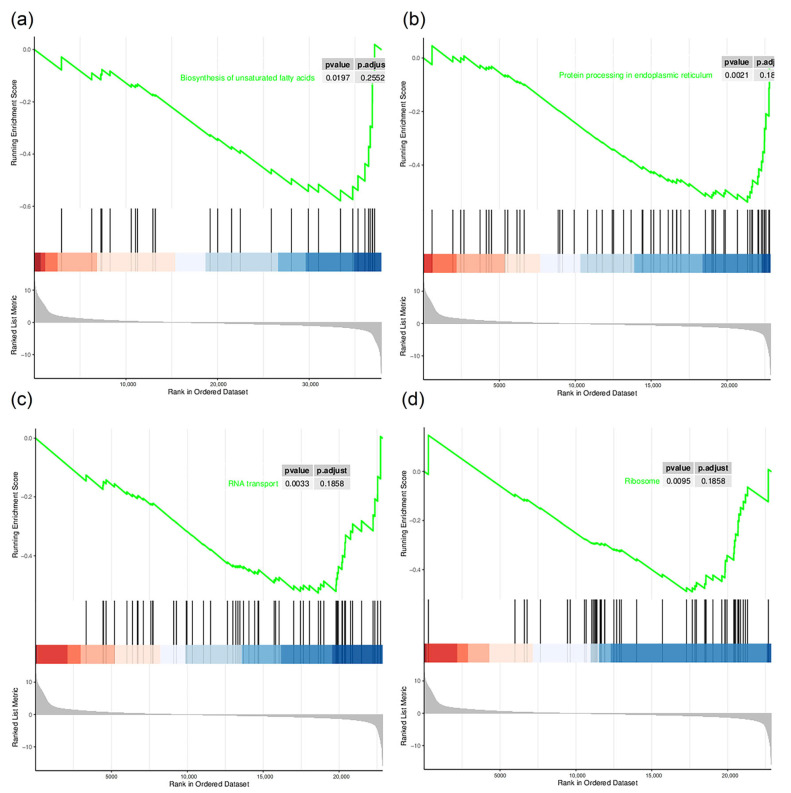
GSEA of downregulated pathways in gregarious armyworms compared with solitary armyworms. The interpretation method of figures is the same as that in Figure 5. (**a**) Biosynthesis of unsaturated fatty acids; (**b**) protein processing in the endoplasmic reticulum; (**c**) RNA transport; (**d**) ribosome.

To elucidate the biological significance of the DEGs, we used Gene Ontology (GO) and Kyoto Encyclopedia of Genes and Genomes analyses (Figure 4). The GO analysis annotated the DEGs in the categories biological processes, cellular components and molecular function. The biological processes, including most DEGs, were cellular process, metabolic process and biological regulation. In terms of cell composition, DEGs were assigned to cell structural entity and protein-containing complex. For molecular function, most DEGs were assigned to the categories binding, catalytic activity and molecular function regulator. The KEGG enrichment analysis showed that in gregarious armyworms compared with solitary armyworms, multiple upregulated genes were enriched in energy metabolism pathways, including pentose and glucuronate interconversions, amino sugar and nucleotide sugar metabolism, the AMP-activated protein kinase (AMPK) signaling pathway and the insulin signaling pathway. Downregulated genes were enriched in protein processing in the endoplasmic reticulum, RNA transport, protein export, purine metabolism, RNA polymerase, pyrimidine metabolism, ribosome biogenesis in eukaryotes, aminoacyl-tRNA biosynthesis and other pathways. The Gene Set Enrichment Analysis (GSEA) based on KEGG can identify metabolic pathways that are upregulated or downregulated as a whole based on all the annotated genes. Among them, tyrosine metabolism, the tricarboxylic acid (TCA) cycle, pyruvate metabolism and protein digestion and absorption were significantly upregulated in gregarious-type armyworms compared with solitary-type (Figure 5), whereas protein processing in the endoplasmic reticulum, RNA transport, the ribosome and biosynthesis of unsaturated fatty acids were significantly downregulated (Figure 6). We found that *M. separata* has two insulin-like receptor (*InR*) genes. One of them (TRINITY_DN1670_c0_g1) was significantly upregulated in gregarious larvae, while the other (TRINITY_DN6759_c0_g1) showed no significant change in expression. 3-Phosphoinositol-dependent protein kinase (*PDK*, TRINITY_DN468_c0_g1) was significantly upregulated in gregarious-type larvae. Significantly, although there was no significant change, three takeout genes (*TO*, TRINITY_DN1529_c1_g1, TRINITY_DN3829_c0_g1 and TRINITY_DN9355_c2_g1) were upregulated in gregarious-type larvae. In conclusion, at the transcriptome level, high population density significantly affected the sugar metabolism, transcription and translation processes of the armyworms. Sequences of the two *InR* genes, the *PDK* gene and the three takeout genes are provided in Table S3.

### 3.3. Effect of Density on Metabolome of M. separata

**Figure 7:**
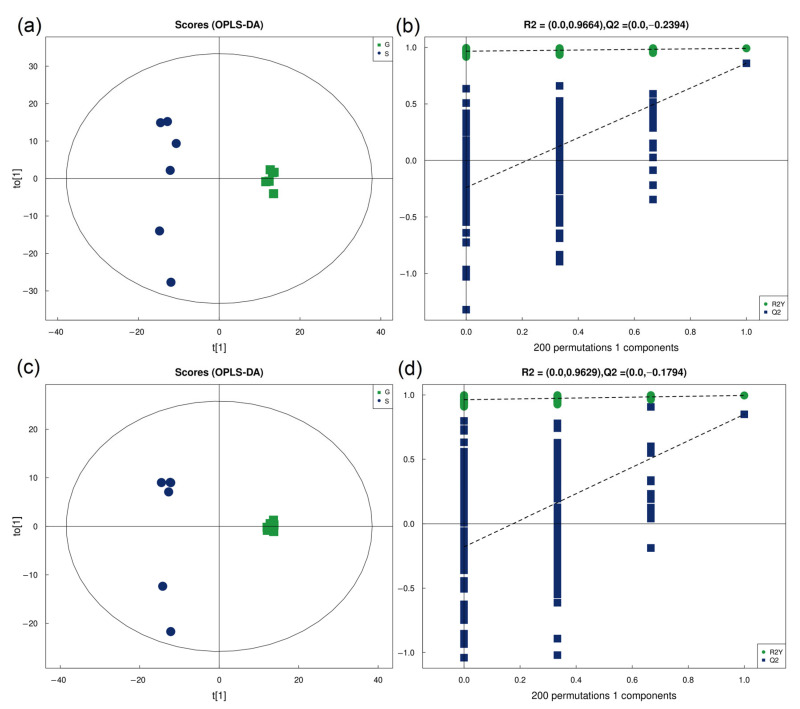
Orthogonal partial least-squares discriminant analysis (OPLS-DA) of the metabolome of gregarious armyworms and solitary armyworms. (**a**) Positive-ion mode score chart; (**b**) positive-ion mode permutation test; (**c**) negative-ion mode score chart; (**d**) negative-ion mode permutation test.

In this part of the experiment, we measured the metabolome of second-day sixth instar gregarious and solitary *M. separata* larvae. In this study, an ultra-high-performance liquid chromatography quadrupole time-of-flight mass spectrometer (UHPLC-Q-TOF MS) was used to identify metabolites of gregarious and solitary larvae. It takes longer for the positive and negative ions to scan separately, and more kinds of substances can be obtained. Therefore, we chose to analyze in positive-ion mode and negative-ion mode, respectively. The quality rating results of the metabolomic experimental data are shown in Figure S2. The test results showed that the instrument functioned well and the experiment had good repeatability. The results of the orthogonal partial least-squares discriminant analysis (OPLS-DA) showed that the two groups were clearly distinguishable, indicating a significant difference between the metabolic profiles of the two types of larvae: positive-ion mode—R^2^X = 0.826 cum, R^2^Y = 0.992 cum and Q^2^ = 0.86 cum; negative-ion mode—R^2^X = 0.79 cum, R^2^Y = 0.996 cum and Q^2^ = 0.849 cum. Cum stands for cumulative. R^2^X represents the model’s interpretation rate to the X variable, and R^2^Y represents the model’s interpretation rate to the Y variable; Q^2^ indicates the model prediction ability. Generally, a stable and reliable model requires a Q^2^ higher than 0.5. Permutation tests indicated that there was no over fitting in the model (Figure 7). After combining the positive- and negative-ion models, 1570 metabolites were identified in the tested samples. All identified metabolites are listed in Table S4 (POS) and Table S5 (NEG). The chemical classification of metabolites is shown in Figure S3.

**Figure 8:**
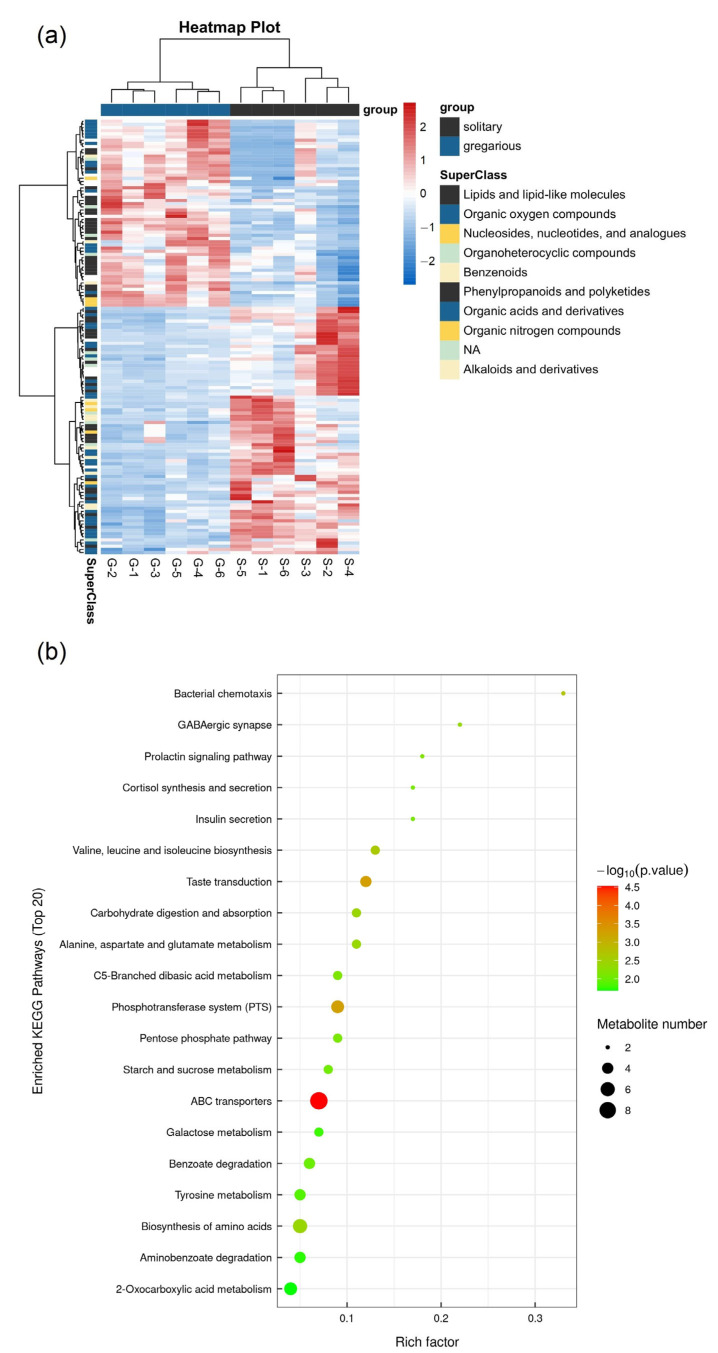
Analysis of differentially accumulated metabolites between gregarious and solitary second-day sixth instar oriental armyworm (*M. separata*) larvae. (**a**) Clustering heatmap: S indicates solitary type and G gregarious type. (**b**) KEGG enrichment bubble chart.

Overall, 137 differentially accumulated metabolites (DAMs) were identified between gregarious armyworms and solitary armyworms, including 59 upregulated metabolites and 78 downregulated metabolites (gregarious type vs. solitary type). A DAM heatmap showed the difference in metabolite abundance between the two types of armyworm larvae (Figure 8a). The abundance of various energy-metabolism-related substances changed. L-glutamine, pyruvate and a variety of sugars decreased in abundance in the gregarious type, while the abundance of AMP, glucose-6-phosphate and a variety of oligopeptides increased. Supporting data for Figure 8a are shown in Table S6. To elucidate the relationships between DAMs and the phase change of armyworms, we introduced the DAMs into the KEGG database, and determined the most relevant metabolic pathways based on the *p* value and pathway impact scores. A large number of DAMs were enriched in ABC transporter pathways, the phosphotransferase system (PTS), carbohydrate digestion and absorption and the pentose phosphate pathway (Figure 8b).

### 3.4. Association Analysis of Transcriptomic and Metabolomic Data

**Figure 9:**
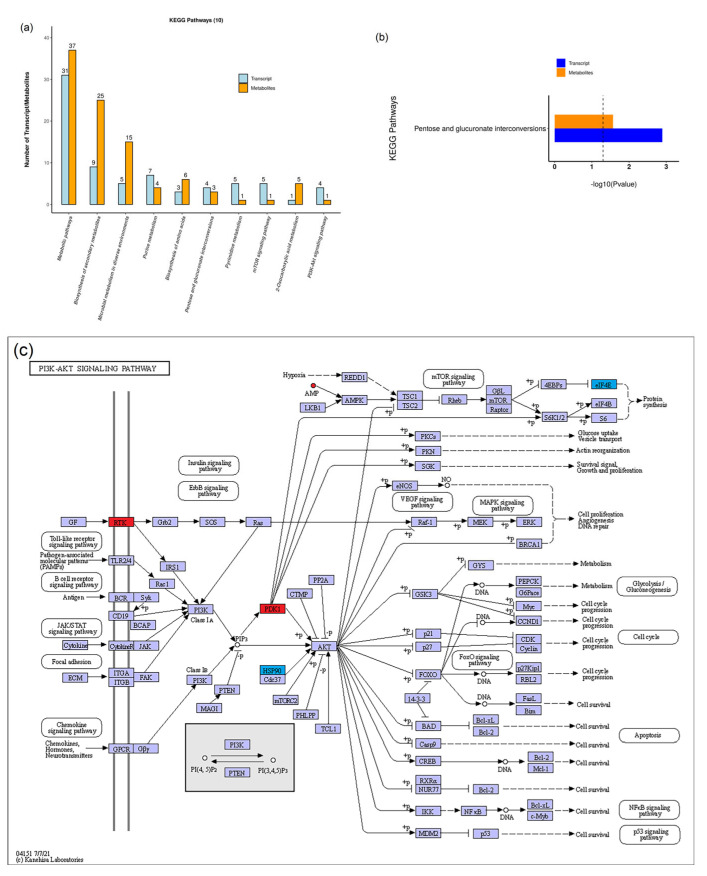
Comparative analysis of transcript and metabolite KEGG pathway annotation. (**a**) The 10 pathways with the largest number of DEGs and DAMs participating together. Each column represents a KEGG pathway, blue represents the transcriptome and orange represents the metabolome. From left to right, the number of genes or metabolites is arranged from high to low. (**b**) Enrichment of DEGs and DAMs in pentose and glucuronate interconversions pathway. (**c**) Map of gene expression and metabolite abundance differences in the PI3K-Akt signaling pathway in gregarious armyworms compared with solitary armyworms. DEGs and DAMs are marked in red and blue. Red indicates upregulation and blue downregulation.

**Figure 10:**
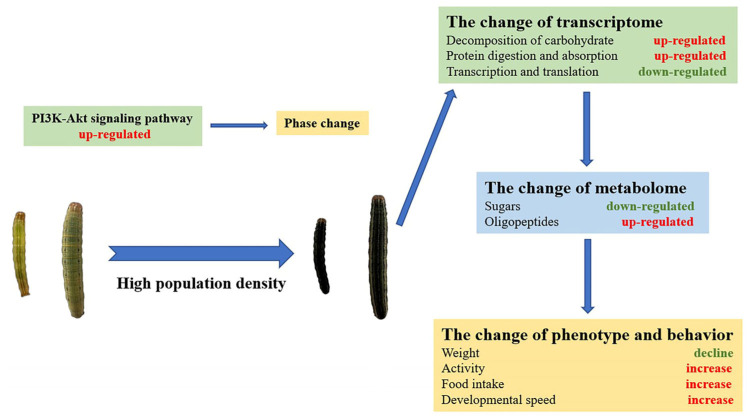
Response of armyworms to high population density.

We integrated transcriptome and metabolome data based on the KEGG database annotation results. The joint analysis of the transcriptomic and metabolomic data shows that several metabolic pathways were shared by DEGs and DAMs (Figure 9a). Pentose and glucuronate interconversions was the only metabolic pathway in which both DEGs and DAMs were significantly enriched (Figure 9b). In this experiment, the expression of several genes and the abundance of metabolites in the phosphatidylinositol-3-OH kinase (PI3K)-protein kinase B (Akt) signaling pathway were found to have changed significantly between gregarious and solitary armyworms, including *RTK*, *PDK*, *HSP90*, *eIF4E* and AMP. The PI3K-Akt signaling pathway can respond to insulin stimulation and promote cell growth, which may play an important role in the phenotypic differentiation of insects. DEGs and DAMs related to the PI3K-Akt pathway are shown in Figure 9c. On the basis of the results of the transcriptomic and metabolomic analyses, the main physiological and molecular divergence between gregarious and solitary armyworms obviously lies in energy metabolism (Figure 10).

## 4. Discussion

Researchers have noticed the relationship between body color and population density of *M. separata* for a long time. We now have some knowledge of the molecular mechanisms that regulate the melanism of noctuid moths. Numerous members of the pyrophosphokinase/pheromone biosynthesis-activated neuropeptide (PK/PBAN) family can induce melanism in armyworm larvae. These neuropeptides exert functions by activating the PBAN receptor (PBAN-R) and subsequent Ca^2+^ influx, which activates cAMP or directly activates downstream kinases [21,22]. The growth blocking peptide (GBP) can also regulate pigmentation on the body surface of armyworm larvae [6]. The signal conduction pathway is initiated by inositol triphosphate (IP3) produced from activated phospholipase-C (PLC), which triggers the flow of Ca^2+^ from the extracellular fluid into the cytoplasm and increases the concentration of Ca^2+^ there, enhancing the expression of tyrosine hydroxylase (*TH*) and DOPA decarboxylase (*DDC*) genes, in turn affecting the body color [23].

For *M. separata*, individual food consumption increases with the increase in population density, which leads to a sharp increase in the degree of damage caused by this pest [18]. This phenomenon has also been reported for *Epirrita autumnata* [24]. We do not know much about the molecular mechanisms behind this behavior pattern. Through transcriptomic and metabolomic analyses measuring gene expression and metabolite abundance in gregarious and solitary armyworms, we can identify the key genes and metabolites that induce changes in the phenotype and behavior patterns of armyworms, providing reference data for scientific methods of pest control. The sixth instar of *M. separata* causes the most serious damage to crops (the food intake during the sixth instar is more than the sum of that during the first five instars). The body color of the larvae on the second day of the sixth instar is stable, and thus is suitable for distinguishing gregarious and solitary types. Therefore, this study used second-day sixth instar armyworm larvae for transcriptomic and metabolomic analyses.

Different insects have one or two genes encoding insulin-like receptors (*InR1* and *InR2*). Many insects with polymorphism, such as *Harpegnathos saltator* and *Apis mellifera*, have two insulin-like receptor genes [25,26]. The insulin-like signaling pathway mediated by InR plays a major role in insect phase change. The *H. saltator* ant queen obtains fertility by regulating the insulin-like polypeptide to induce mitogen-activated protein kinase (MAPK) phosphorylation, and delays aging by blocking the PI3K-Akt pathway [25]. The two *InR* genes of BPH can control the differentiation of its wing form by regulating the transcriptional activity of FOXO. InR1 of BPH can activate the PI3K-Akt signal cascade to cause long-winged morph, while the expression of *InR2* can block the insulin-like signaling pathway to produce short-winged morph [14]. Akt can be activated in several ways, including the phosphorylation of a specific threonine by PDK and the phosphorylation of a specific serine by the target of rapamycin (TOR) kinase and the rictor protein [27]. The activated Akt translocates to the nucleus and phosphorylates FOXO at three conserved residues. The phosphorylation of these residues decreases the affinity of FOXO for DNA and enhances its binding with the 14-3-3 protein, which mediates the transfer of FOXO to the cytoplasm and decreases its transcriptional activity, thus triggering various cellular reactions [28].

The GSEA showed that the tyrosine metabolism pathway of gregarious armyworms was significantly upregulated compared with that in solitary armyworms. Tyrosine is a precursor of melanin. DOPA (L-3,4-dihydroxyphenylalanine) is produced by the action of tyrosine hydroxylase [29]. DOPA can be transformed into dopamine by DOPA decarboxylase, and melanin is then generated after a series of complex processes [30]. The activation of the PI3K-Akt signaling pathway can promote the proliferation of melanocytes and the synthesis of insect melanin [31,32]. These phenomena indicate that high population density may affect the insulin-like signaling pathway, change the expression of key genes in the PI3K-Akt pathway and thus regulate the phase change of armyworms.

Glutamine is considered to be the fuel of the immune system [33]. In adverse circumstances, glutamine is consumed as an energy source, especially in immune cells [34]. The decreased abundance of glutamine in gregarious armyworms indicates that overpopulation exerts survival pressure on the armyworm. Increased density of armyworms will intensify their competition for food and living space. The acceleration of food digestion and energy metabolism can promote an armyworm to ingest more food and grow faster, thus making it dominant in intraspecific competition. Research on locusts has shown that the difference in flight ability between solitary and gregarious locusts is caused by a change in energy metabolism [35]. A difference in energy metabolism may also exist between gregarious and solitary armyworms and cause different behavior patterns.

Enrichment of glycometabolism-related pathways suggests that gregarious armyworms may accelerate the consumption of carbohydrates. Metabolomic analysis revealed that two key factors in energy metabolism—AMP and glucose-6-phosphate—were more abundant in gregarious armyworms than in solitary armyworms. AMP is associated with the adenylate kinase reaction and activates AMPK to maintain the homeostasis of cellular energy metabolism [36]. Glucose-6-phosphate is an essential intermediate in energy metabolism. In the first step of glycolysis, hexokinase catalyzes the phosphorylation of glucose to form glucose-6-phosphate [37]. Under the action of glucose-6-phosphate dehydrogenase, glucose-6-phosphate is dehydrogenated and converted to glucose-6-phosphate lactone, which is the first step of the pentose phosphate pathway [38]. The changes in expression of various genes and the high abundance of AMP and glucose-6-phosphate in gregarious armyworms indicate that the rate of carbohydrate decomposition in the gregarious type is significantly increased. The change in carbohydrate content in the gregarious type supports this view: among the 137 DAMs, the abundance of most sugars was lower in the gregarious type, including 1,4-D-Xylobiose, D-fructose, D-mannose, D-ribose and maltose.

The abundance of pyruvate was significantly decreased, and pyruvate metabolism was significantly upregulated in gregarious armyworms compared with solitary armyworms. This can also be taken as evidence of accelerated carbohydrate decomposition. Pyruvate is the final product of glycolysis. It can be oxidized to form acetyl CoA in aerobic conditions, enter the TCA cycle and accomplish the aerobic oxidation of glucose for energy supply [39]. Pyruvate is also the raw material for fatty acid synthesis [39]. The downregulation of fatty acid synthase (*FAS*, TRINITY_DN8931_c0_g1) and of the biosynthesis of the unsaturated fatty acids pathway in gregarious-type armyworms indicate that the rate of fatty acid synthesis is decreased in the gregarious-type compared with solitary-type armyworms. Therefore, the additional pyruvate consumed by the gregarious type may not be used for synthesizing fatty acids, but is oxidized to acetyl CoA and then to CO_2_ and H_2_O through the TCA cycle and oxidative phosphorylation.

Increased energy consumption will accelerate the starvation of gregarious armyworms, and starvation induces the expression of takeout genes [40]. The takeout protein plays an important role in regulating the feeding behavior of insects. This protein can improve the appetite of Drosophila and enhance its movement ability when starving [41]. Therefore, the overeating behavior of gregarious armyworms may be upregulated by the increased expression of takeout genes. The fact that we did not observe significant upregulation of these genes in our experiments may be caused by tissue-specific expression and/or sensitivity to the external environment. Starvation treatment can rapidly upregulate insulin-like signaling pathway related genes in *Bombyx mori*, including *InR* and *PDK* [42]. This is consistent with the discussion above.

Significant differences in protein metabolism were found between the two types of armyworm larvae. The significant downregulation of several RNA- and protein-synthesis-related pathways indicated that both transcription and translation processes were inhibited in gregarious-type armyworms. Quantity distribution of DEGs also shows this (481 and 667 genes were up- and downregulated, respectively, in gregarious-type armyworms compared with solitary-type). Previous studies found that the dry weights of gregarious armyworm larvae and pupae were significantly lower than that of the solitary type [6,43]. Protein is the most abundant organic substance in organisms. Therefore, a decrease in protein synthesis can explain this phenomenon. In the case of insufficient energy supply, organisms may achieve energy redistribution by inhibiting the biogenesis of ribosomes, so as to maintain body health [44]. Energy consumption of armyworms increases in response to high-population-density stress, and it may be beneficial to conserve energy by downregulating the expression of noncritical genes and reducing the synthesis of noncritical proteins.

Several serine proteases were significantly upregulated in gregarious armyworms compared with solitary armyworms, and the GSEA showed that protein digestion and ab-sorption were upregulated in the gregarious type, which means that gregarious armyworms have greater dietary protein digestion ability. Increased protease activity and digestive capacity of gregarious insects have been reported in a variety of species, such as *Lymantria dispar* [45]. Therefore, we hypothesized that the voracity behavior of gregarious armyworms may be related to both their rapid consumption of carbohydrates and enhanced protein digestion ability. Proteases break peptide bonds to digest proteins into amino acids and oligopeptides. These small molecules can be absorbed by insects as energy materials or as raw materials for new protein synthesis [46]. Eleven upregulated dipeptides or tripeptides were detected by metabolomic analysis in gregarious-type armyworms compared with solitary armyworms. These oligopeptides are most likely products of protein digestion. Food-protein-derived peptides possess many other beneficial physiological functions while also serving as nutrients [47]. For example, Ile–Pro–Ile (diprotin A) and Val–Pro–Leu (diprotin B), dipeptidyl peptidase-4 inhibitors, increase the insulin level and lower blood glucose in animals [48,49]. The increased abundance of these tripeptides is consistent with the changes in carbohydrate metabolism and the insulin-like metabolism pathway that we observed in gregarious-type armyworms.

## 5. Conclusions

On the basis of analysis of transcriptomic and metabolomic data, this study summarizes differences in gene expression and metabolite accumulation in second-day sixth instar larvae of gregarious and solitary *M. separata*. DEGs and DAMs were significantly enriched in carbohydrate-decomposition-, transcription-, translation-, and protein-digestion-related pathways, which indicates that changes in energy metabolism are key to the phase change of *M. separata*. These results broaden our understanding of the physiology of *M. separata* and will help us formulate more scientific strategies for control of this pest.

## Data Availability

Raw data were deposited in the NCBI Short Read Archive (SRA) database, and the accession numbers are SAMN31828535, SAMN31828536, SAMN31828537, SAMN31828538, SAMN31828539 and SAMN31828540.

## Supplementary Materials

The following supporting information can be downloaded at https://www.mdpi.com/article/10.3390/insects14010068/s1, Table S1: Primers for qPCR; Table S2: List of all unigenes; Table S3: Sequences of the two *InR* genes, the *PDK* gene and the three takeout genes; Table S4: List of all metabolites—POS; Table S5: List of all metabolites—NEG; Table S6: Supporting data for Figure 8a; Figure S1: Comparison of RNA-Seq and qPCR results; Figure S2: The quality rating results of the metabolomic experimental data; Figure S3: The chemical classification of metabolites.
