# Supplementary material for: Multi-Omics Reveals the Effect of Population Density on the Phenotype, Transcriptome and Metabolome of Mythimna separata
Source: Insects. 2023 Jan 10;14(1):68. doi: 10.3390/insects14010068 (PMC9861010; doi:10.3390/insects14010068)
Supplement: Supplementary file 1 [file insects-14-00068-s001.zip › Figure S1 Figure S2 - conversion.pdf]

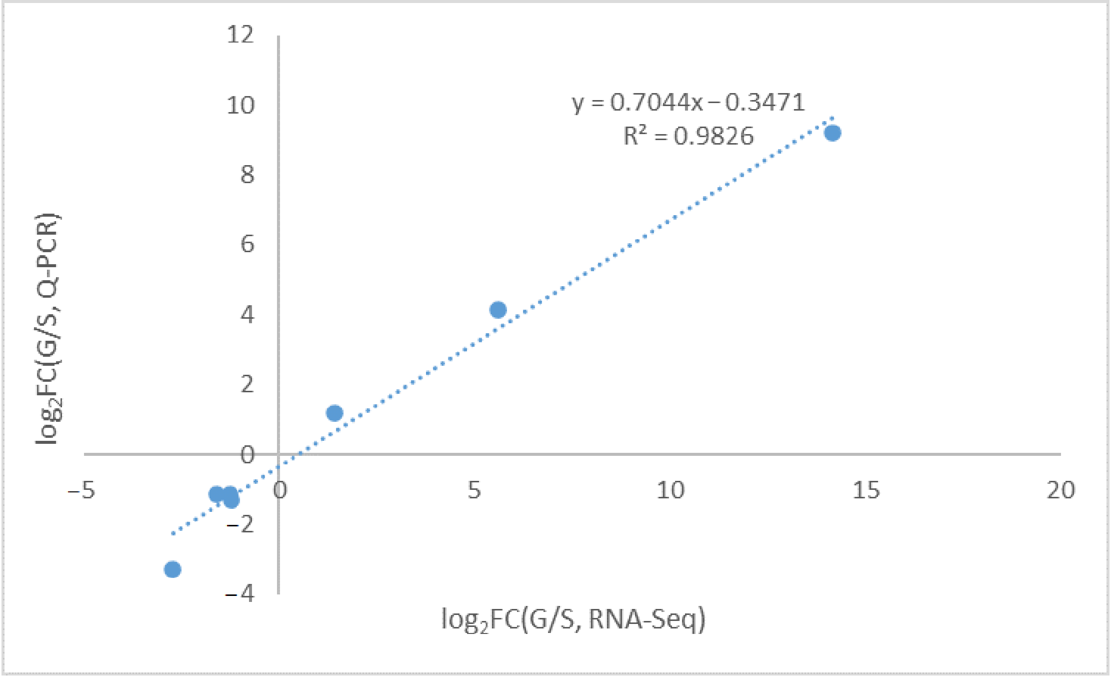


**Figure S1.** Comparison of RNA-Seq and qPCR results. The horizontal axis is the result of RNA-Seq, and the vertical axis is the result of qPCR. The number on the coordinate axis represents the log2 value of each gene expression ratio of gregarious and solitary armyworms.

| Gene | $log_2$FC(G/S, RNA-Seq) | $log_2$FC(G/S, qPCR) |
|---|---|---|
| TRINITY_DN11213_c0_g4 | −1.23264 | −1.29826 |
| TRINITY_DN1026_c5_g1 | −1.61120 | −1.13386 |
| TRINITY_DN11743_c0_g1 | −1.26588 | −1.12059 |
| TRINITY_DN876_c3_g1 | −2.72646 | −3.29073 |
| TRINITY_DN2583_c0_g1 | 1.39784 | 1.16565 |
| TRINITY_DN1017_c0_g1 | 5.61830 | 4.13641 |
| TRINITY_DN1037_c0_g2 | 14.1709 | 9.21950 |

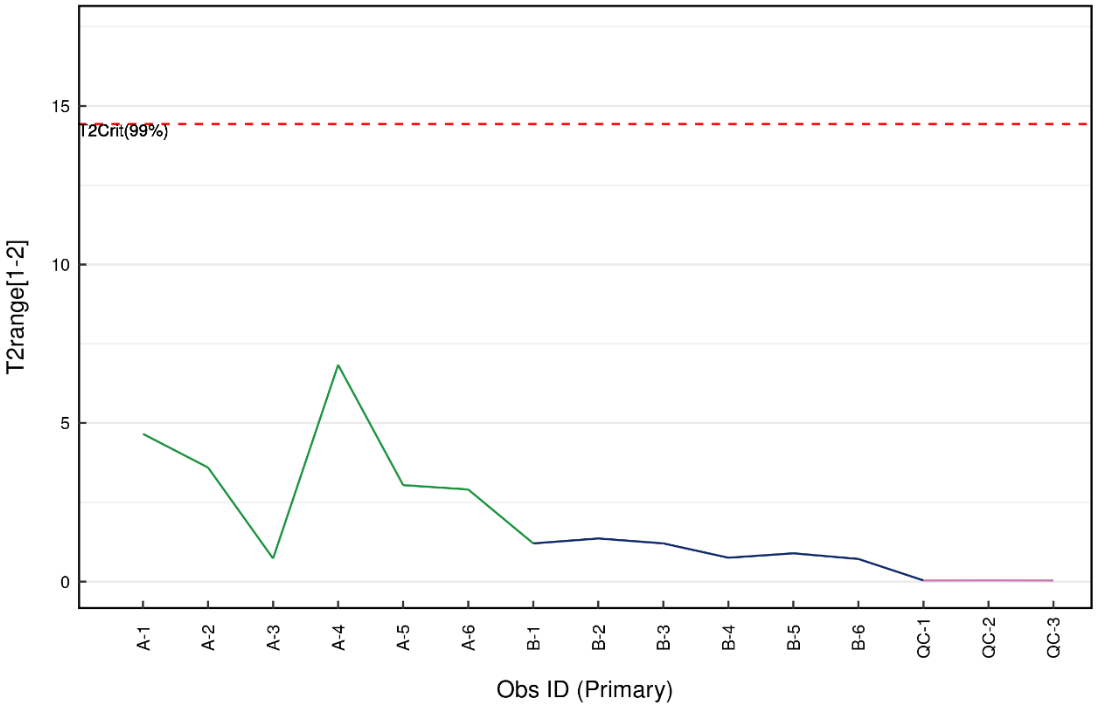


**Figure S2A**. Hotelling T2 range line plot of negative ion mode population samples.

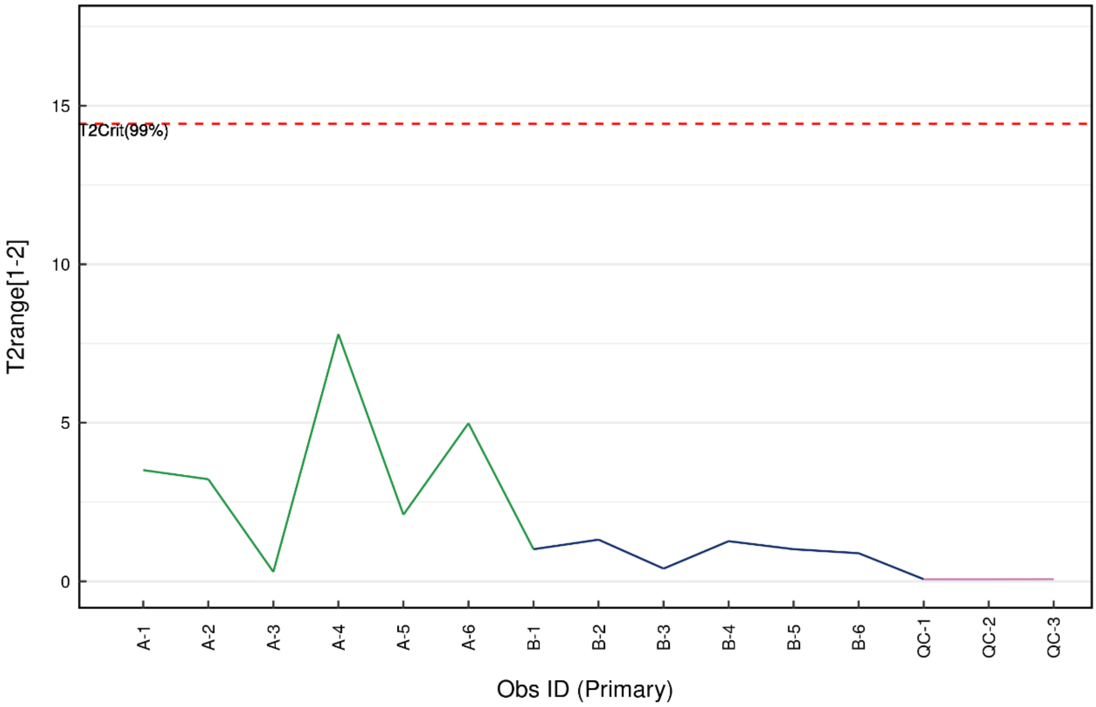


**Figure S2B**. Hotelling T2 range line plot of positive ion mode population samples.

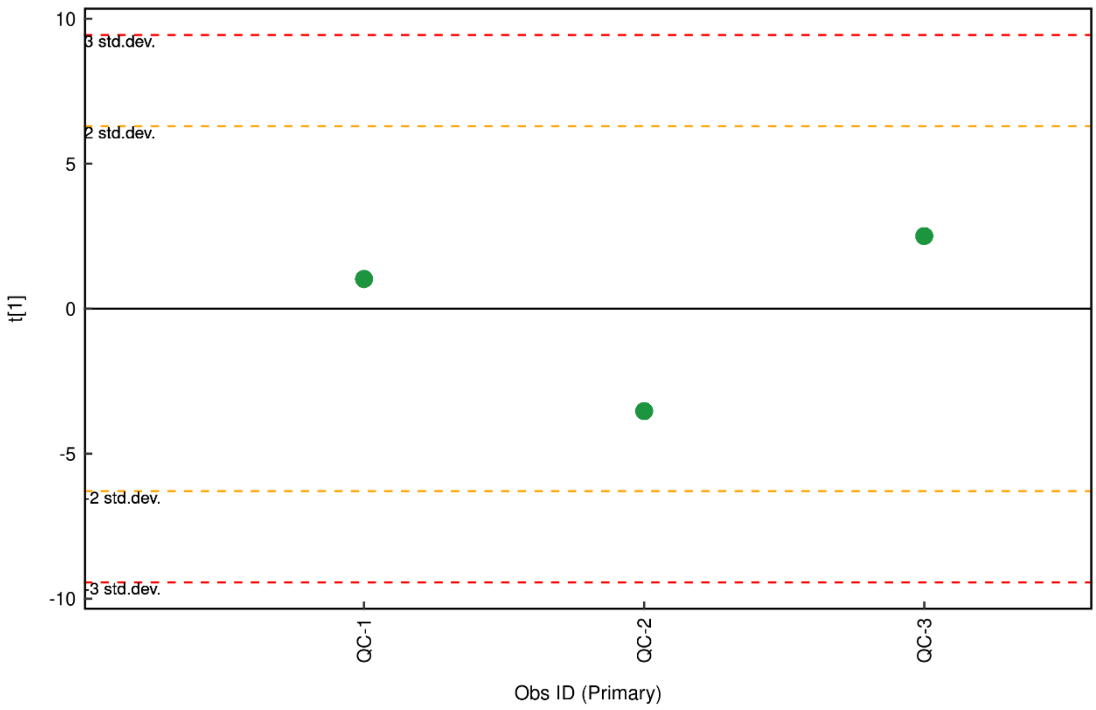


**Figure S2C**. MCC map of QC samples in negative ion mode.

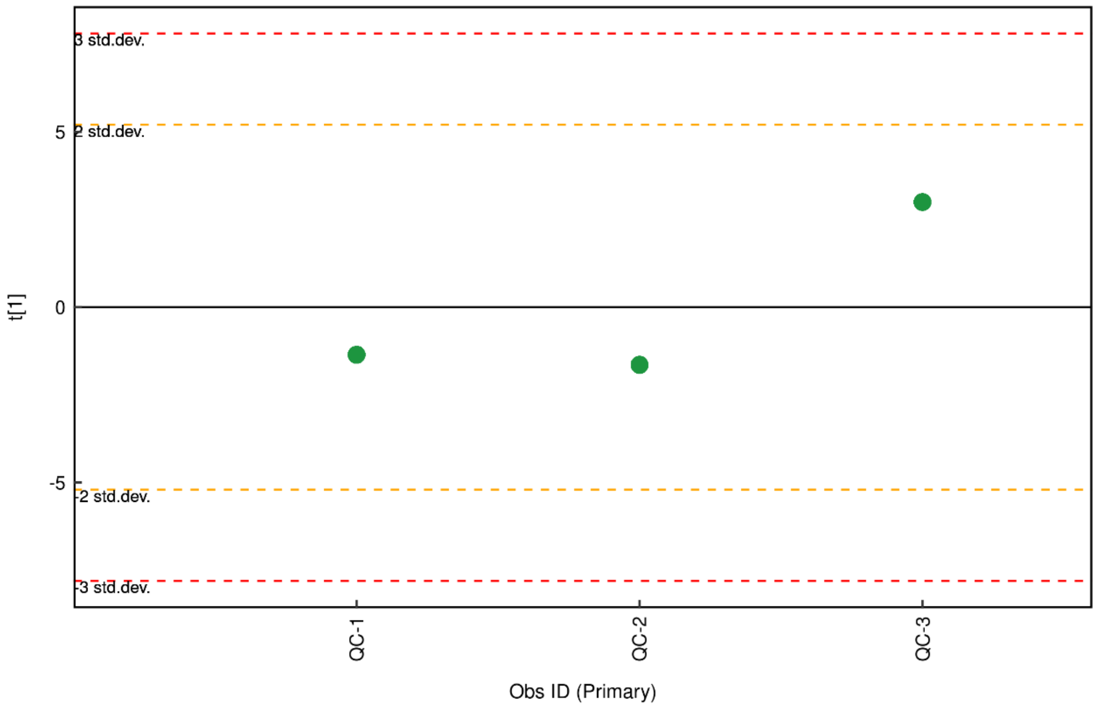


**Figure S2D**. MCC map of QC samples in positive ion mode.

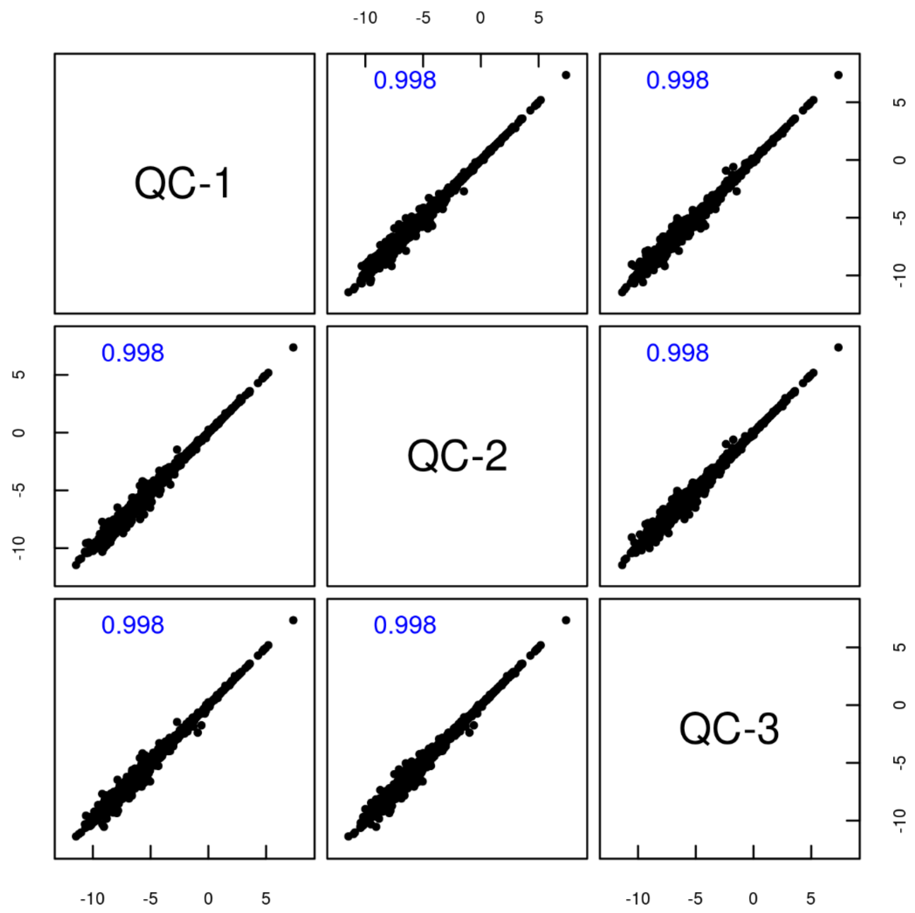


**Figure S2E**. Correlation map of QC samples in negative ion mode.

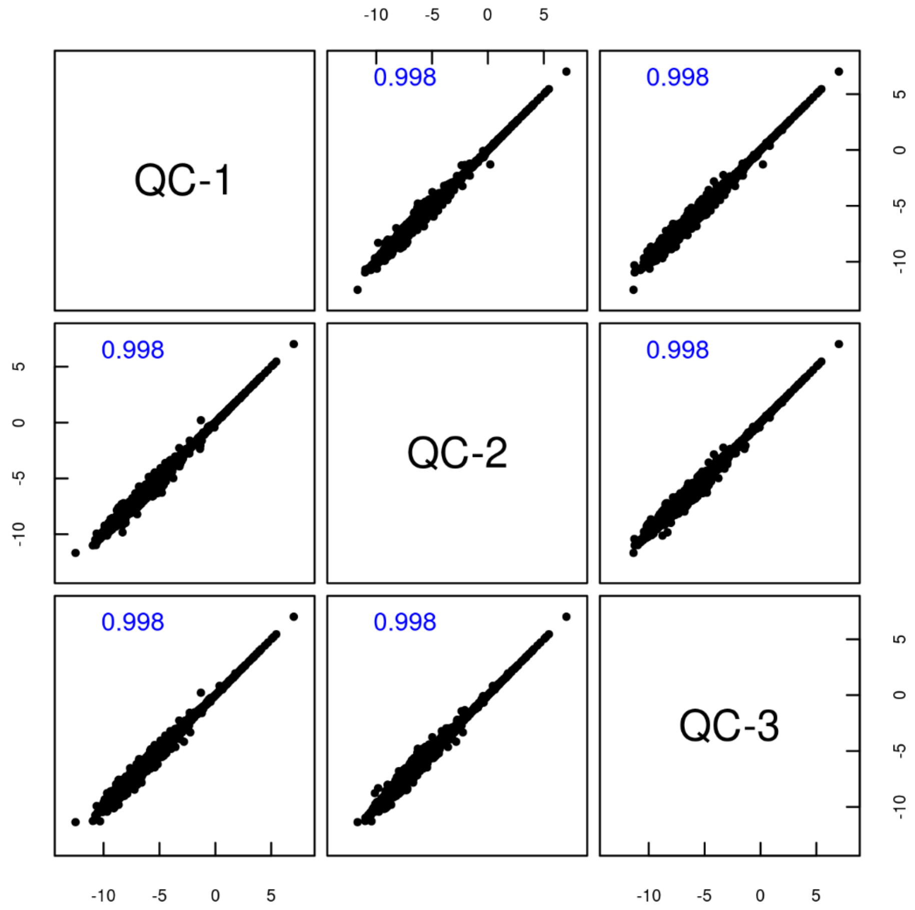


**Figure S2F**. Correlation map of QC samples in positive ion mode.

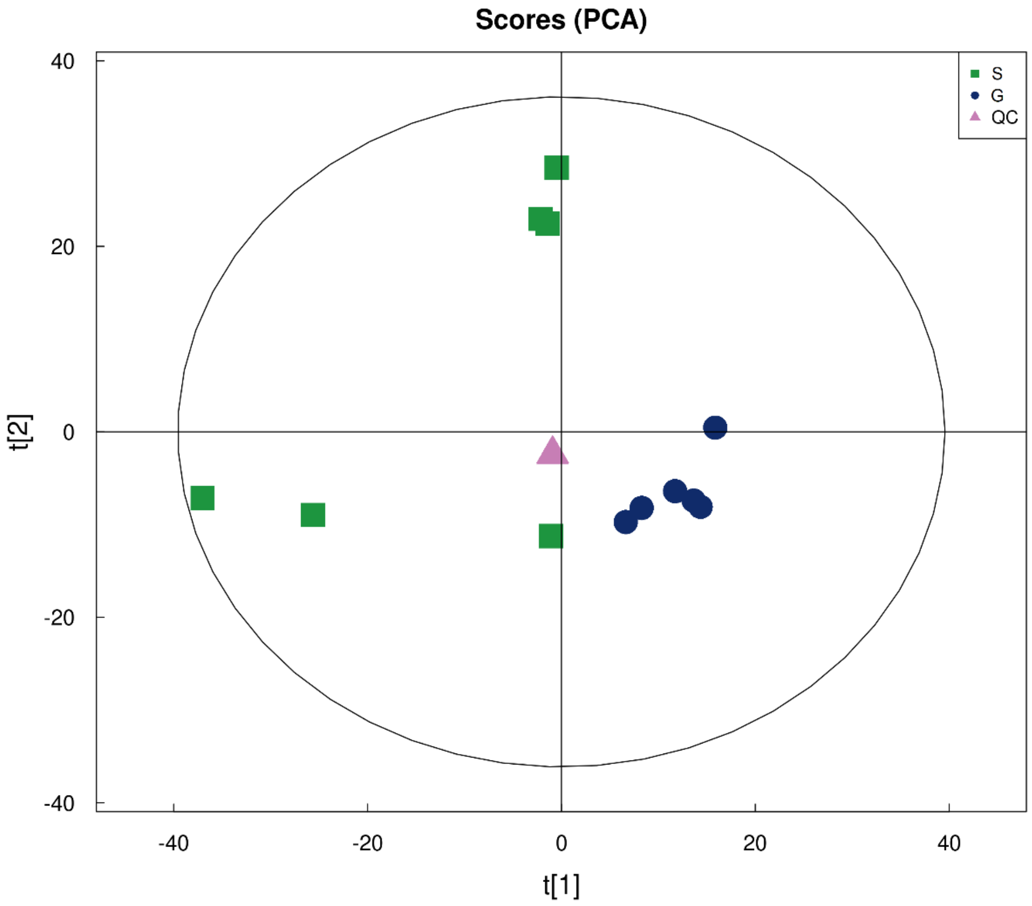


**Figure S2G**. PCA analysis of negative ion mode population samples.

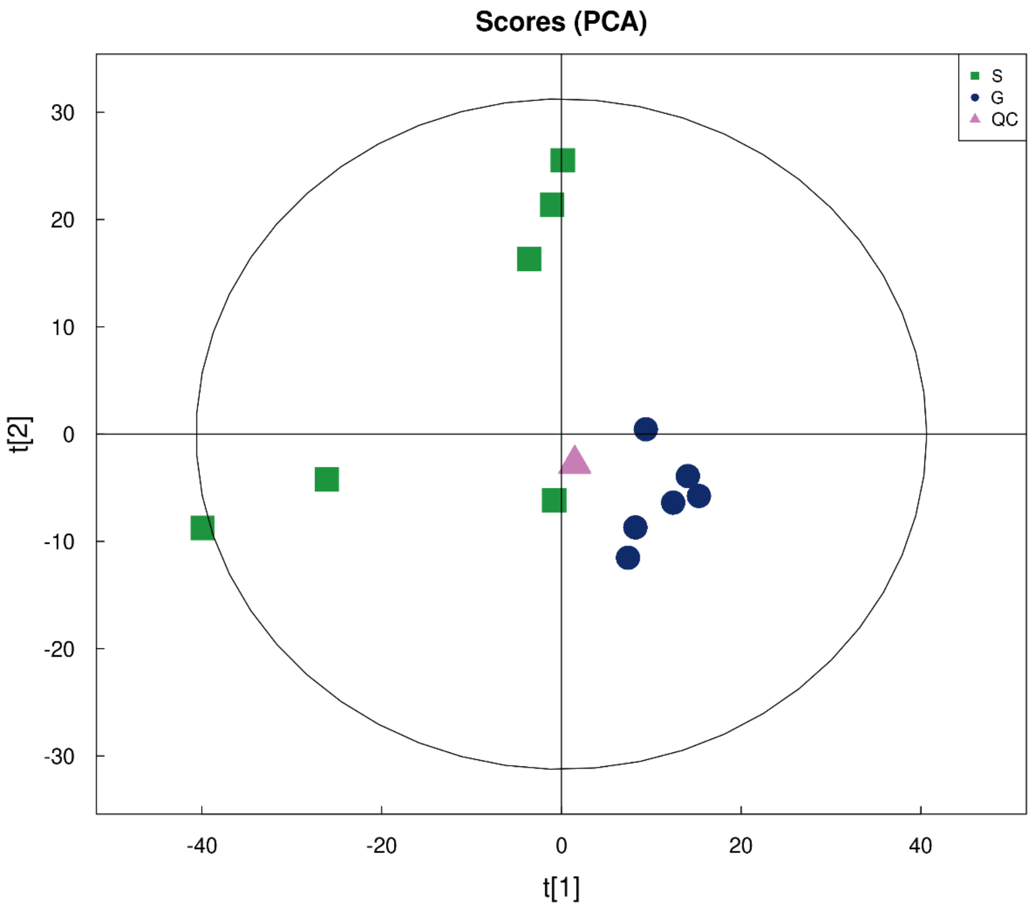


**Figure S2H**. PCA analysis of positive ion mode population samples.

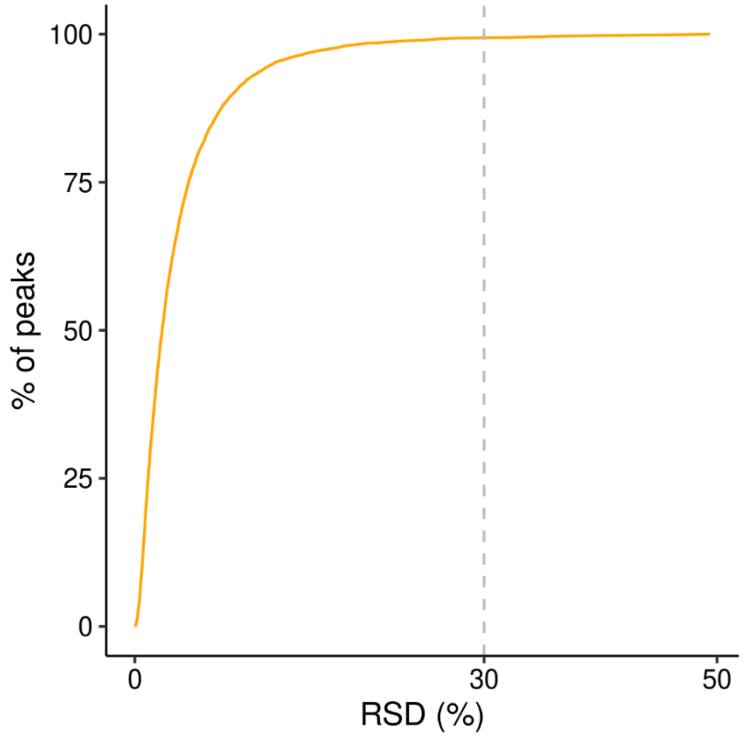


**Figure S2I**. Relative standard deviation of QC samples in negative ion mode.

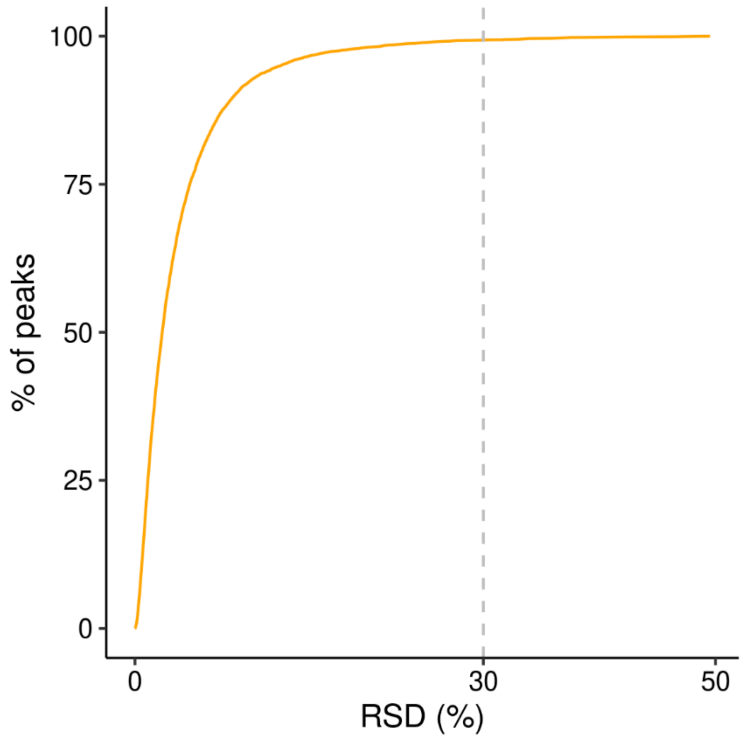


**Figure S2J**. Relative standard deviation of QC samples in positive ion mode.
