# Supplementary material for: Multi-Omics Reveals the Effect of Population Density on the Phenotype, Transcriptome and Metabolome of Mythimna separata
Source: Insects. 2023 Jan 10;14(1):68. doi: 10.3390/insects14010068 (PMC9861010; doi:10.3390/insects14010068)
Supplement: Supplementary file 1 [file insects-14-00068-s001.zip › Table S1.pdf]

**Table S1** Primers for qPCR

| | |
|---|---|
| *TRINITY_DN11213_c0_g4*-F | CGCTCTGACAAACCCCGT |
| *TRINITY_DN11213_c0_g4*-R | CCAGCCAATCAACCTCCA |
| *TRINITY_DN1026_c5_g1*-F | ACATTTGTTGCGTCTGGC |
| *TRINITY_DN1026_c5_g1*-R | CACGGTTTTGACTTGCCT |
| *TRINITY_DN11743_c0_g1*-F | GTCACTTGTGCGTTTTTCGT |
| *TRINITY_DN11743_c0_g1*-R | GTGTTCGCTTTCTCCCTTTT |
| *TRINITY_DN876_c3_g1*-F | TCTTTCTCACTTCTTGCTTGG |
| *TRINITY_DN876_c3_g1*-R | ATGTTTCGTTACGGGTTGGTT |
| *TRINITY_DN1017_c0_g1*-F | CTTTGCTGGCGTAACTGGT |
| *TRINITY_DN1017_c0_g1*-R | AGGGAGGTTGTCGTGGGGA |
| *TRINITY_DN2583_c0_g1*-F | CAGATGTTGGCAATGGGCTT |
| *TRINITY_DN2583_c0_g1*-R | CTTCTTGGGGTTGACGGGG |
| *TRINITY_DN1037_c0_g2*-F | GGGTAGATAGCCCATACGCAT |
| *TRINITY_DN1037_c0_g2*-R | GGAGAGGGAGACCATCACTGT |
| *β- Actin*-F | CCAACGGCATCCACGAGACCA |
| *β- Actin*-R | TCGGCGATACCAGGGTACAT |
