# Supplementary material for: Multi-Omics Reveals the Effect of Population Density on the Phenotype, Transcriptome and Metabolome of Mythimna separata
Source: Insects. 2023 Jan 10;14(1):68. doi: 10.3390/insects14010068 (PMC9861010; doi:10.3390/insects14010068)
Supplement: Supplementary file 1 [file insects-14-00068-s001.zip › Table S3.pdf]

InR1<br>TRINITY_D<br>N1670_c0_g<br>1	>TRINITY_DN1670_c0_g1<br>CGGCAGGCCCCGCGCTCTCCGCTTCCCTCCCTCAGTCGTTCGCGCGCGTCGTTTGTGAT<br>AACTCGTCTGCACGTTATCCGCTGTTTGCCGCGTATTATGTTTACAATAGTGATGTTATT<br>GTTGTGGTTATTATAAAAATACACAATTCAAGTGTTTAGTGCTGTGTGACAAATTGAAA<br>TATTGTACTGAAGGATCTGTTGAATATAAATAAACTGTGGATCGCAAGGAGCAGACAA<br>GCACGGCAGGCACCGAGCTCAGCTCGGGTCATGTGGTCAGCGGGCCAGGGGCGCGG<br>CACGGCGTGATGCGGCGCGCGCGCAACAGCCTCGCATGGCCGGTGCTGGCGTGCGCG<br>CTGCTCTCAGTATGCGCGTGCGCCGATGCCATCGGCGAGAATGGAATATGCCCTTCAA<br>TGGACATAAGGGGCGATTTATCCGCCCTGAAGAATCTGAAGAACTGTCGAGTCATCGA<br>AGGCCAGCTCAGCATCGTGCTGATGGAGATGGCCACGCCCAAGGACTACGAGAACAT<br>GTCCTTCCCGCTTCTAAGAGAGGTCACCGATTATGTTCTGATGTACAGGACGAAAGGT<br>TTGCAGAATCTCGGCAATCTGTTCCCCAACCTCTCCGTGATACGCGGGATGCAGCTCTT<br>CAAGGATTTCGCGCTCGTTATATTCGATAATGAGCATTTGGAATCCCTGGGTCTTCGTTC<br>TTTGATGAAGATCGAGCGGGGTGGTGTGCGGATACAACAGAACGACAGGCTCTGCTA<br>CACAGACACCATCGACTGGACCCGGATCACCACGGACCATGCCGACAATGTTATAAG<br>GATGAACTACGACACGCGACTGTGTGGGCTGTGCCCGAATGCGCAGAGTCGTGTCCA<br>GGACCATCGGCTGTCGCACTTGCAGTGCCCGTCCGATGCGAACGACCGCTTGCTCTGT<br>TGGGACGACAAGCACTGCCAGAAACTGTGTTCGACGCAATGCGGCGACCACGGCTG<br>CCTGGACAACGGCACTTGCTGCCACCCGAGCTGCCTGGGCGGCTGTTCGGGGCCCAC<br>CTCCCGGGACTGCCACGCCTGCCGCAACTTCTCCTTCGGAAACGGCAGCGAGCGCAT<br>ATGCCTGGACTCCTGCCCTGCTAATGGCACTTTTCAGTTGTTCCATCGCTGTGTGACGG<br>AGCAGGAGTGCCGCGACACCCCGCCGCCCCTCACCGCCGACCCGAGGCCCCGGAGG<br>TACAAGATCTCCGGCAACCGCTGCGTCTACGACTGTGACATCGGCTACATGGAGGTCG<br>GCGATCCTAAAAACGCGACATGTGTGAAATGTTCCCCGTCGGGCTGCAGAAAGGACT<br>GTCTGGGAGACAAGATCGACTCGGTGGCGGCGGCCGAGAAGTACCGCGGCTGCACG<br>CACGTTACTGGCAACCTCGAGATCAGCTTGCGCGCCTCTGGAGGTAACACGTTGGCAC<br>TGTTAGAAGAGTCTTTGGGTGAAATCCGCGAGATTACTGGCTCGTTGTCGGTCATACG<br>AGCTTATCCACTGGTGTCACTGATGTTTCTGAAAAACCTGAAGAAAATCACAGCATTG<br>CCCCATTCTGAATCGGACATGAAGAAAAATAGAGGTCAAGTATTACACATATTCAACA<br>ATCCGAACCTGGAGTTGTTGTGGGACTGGACGACTCACGGCGACATCGACATCTCCG<br>GCGGCTCGCTGTACATACACCTGAACCCCAAGCTGTGCTACTACACGCAGCTGCTGCC<br>GCTCAAGAACATGACGAGGAACCCCCGCTCCAACTTCACTGACTTGGAAGTTTCCGA<br>AGACAACAACGGGAATCAGGCCTCTTGTCTACCGGACAAGTTGCACTTGCAAGTGAG<br>TCAGTACCACCCCATGACGGTGGTGCTGCAGTGGAAGCGCTATTGCCCCGAGGACATC<br>CGCAAGCTGCTCGGCTACTCCGTGTACTACATCGCCACCAAGCCCAACCAGAACGTC<br>ACGCTCTACGAGCAGAGGGATGTCTGCTCCGATATATGGAGTGTTCAGGATATATCAG<br>CGGATGAAGTACATAATGAATCAAGTACACTTTTAACTACCAGCCAACACGACATTCT<br>TCAGAATCCATGCCTTAATACGCAACCGAGTTTCTACGTGTTGGCACATTTGAGTCCTT<br>ACACGCGCTATGCAGCGTATTTGAAGACTGTTACCACTTCACAGGAGAAAAAGGGTG<br>CACAAAGTTCCATCATATACTTCACAACATTACCTGGGAGACCGAGTGAACCTCAGAG<br>CTTGACGGTGGAGGCGCTGACGCCGCACTCGGTGTCCATCCGCTGGGCCGAGCCGCT<br>GCTGCCCAACGGCACCATCGTGCTGTACCACGTGGCCGTGCAGGCCAGCAGCTACAA<br>CCTGCCGCAGCTGCTGGCCGGCAACCCCAACTACTGCGCCAACCCGAGTGCCTTGGC<br>GAATATGATCACTGGTCGAACAGAGGAGTCAAGTACGAACACTAAGAAAAAAGAGA

	CGACCGGTGACGTCACTAACGGGTCCTGCTCCTGTAAGGAAGAGGAGAAGCAGACC<br>ACGAGGTTCAACTCCCGATCGGAGTGGGAGCGGGAGGAAACCATCACCTTTGAGAAT<br>GACCTGCAGAATAGGGTGTACGTAAAGACGACTGAGAGACAGAAGCATTCAAAAAG<br>CAGAGTAAAACGATCTCAGAACCACTACATGAACAGCATGCTTGTGAAAGTGGCCGA<br>AAGCATCGAGCCCAAGTTTGGTCAAAACTATACCAATACCACCGATGCGGAAGGATAT<br>GTGGAGTCACTGTACTTCGAGCTAAGCGGCGACACGAAGTCGCTGACGGTGACGAAC<br>ATGCGGCACTTCACGTGGTACACGGTCAACGTGTACGCGTGCCGCGCCAAGCACGAG<br>AAGGAGCAGCCCGAGGTGTACGAAGCGACGTGGTGCTCCAAGCACAGCGCTTATAAC<br>AGCTTCCGGACTCTGGAATTAGTGAACGCGGATATAGTGCACAATTTGCAAGCAGAAG<br>TGATCGTGTCGAACAAGACGACAGCCGAAGTGAACGTGACGTGGAAGCCGCCCAAG<br>AACCCCAACGGGTTCGTGGTCGCGTACACCGTGCACTACTATCGCGTGGAAGACAGC<br>TTGGCGGGGCAGGACGTGGGGCTGCAGAGCTGCATCACGGCGGACGACTACGCGCA<br>GAACGGGTTCGGCTACACGCTGCGCAACGTGGCGCCCGGCAACTACAGCGTCGAGGT<br>CACGCCCATCACCGCCGCCGGCGCCGGCAACGCCTCCGCCGTCTACCCGCCCATCTTC<br>ATACCGGAGAGAAAATCGGCCGCAGCTTACGACTGGATCTGGGGCGTGGTGGGCGGC<br>TGCGTGATCATGCTGCTGGTGCTGGGCGGCGGCATCTGGTACGCGCGCCGCGGCCTGC<br>TGCCGTCCGCCGAGGCCAACAAGCTGTTCGCCAGCGTCAACCCCGAGTACGTGTCCA<br>CCATGTACGTGCCCGACGAGTGGGAGGTGCCGCGCACCGCCGTCGAGTTCGTCCGCG<br>AACTGGGCCAGGGCTCCTTCGGCATGGTGTACGAAGGTCTTGGGAAAGGTATAGAAA<br>AAGGGAAACCCGAAACTCGGTGCGCTGTCAAAACTGTCAACGAGCACGCTACTGATC<br>GGGAGCGTCAAGAGTTCCTGAACGAAGCGTCGGTGATGAAGGCGTTCGACACGTTCC<br>ACGTGGTGCGGCTGCTGGGCGTGGTGTCGCGCGGACAGCCCACGCTCGTCATCATGG<br>AGCTCATGGAGTTCGGCGACCTCAAGACCTACCTGCGCTCGCACCGCCCCGACGCCG<br>AGGCCTCGCTGCCGCGCAAGGGCGGCGACGAGCCGCCCACGCTGCAGAACATACTG<br>CAGATGGCTATTGAAATAGCCGACGGTATGGCTTACCTATCGGCCAAAAAATTCGTGC<br>ACCGCGACCTCGCGGCGCGCAACTGTATGGTCGCCGGAGACCTCACCGTGAAGGTCG<br>GGGACTTCGGCATGACCAGAGACATCTACGAGACTGACTACTATAGGAAGGGCACGA<br>AGGGCCTGCTGCCAGTGCGCTGGATGAGCCCGGAGAGCCTTAAAGATGGAGTGTTCT<br>CGAGCAGTAGCGACGTGTGGAGTTACGGCGTCGTGCTGTGGGAGATGGCCACGCTCG<br>CCATGCAGCCCTACCAGGGGCTGTCGAACGAGCAGGTGGTGCGCTACGTGGTGGAGG<br>GCGGCGTGATGGAGCGGCCGGAGCACTGCCCCGACCGCCTGTACGAGCTCATGCGCG<br>CCTGCTGGGCGCACCGCGCGGCGGCCCGGCCCAGCTTCCTGCAGCTCGTGGCCGACC<br>TCGTGCCCAGCTCGCAGCCGCACTTCCGCACGCGCAGCTTCTTCCACACGCCGCAGG<br>GCCAGGAGATGTACAAGCTGCAGCGCACGGCGCTAGAGGAGGAGCAGGAGCTGGCG<br>GAGGTGAACGTGGGCGCCGTGGCCACGGGCTCGGGCTCCAACCTGTTCGGCGTGTCG<br>GGGCGCCTCGCCTCCTGGGTGCGCGAGCTGTCGTCGCTGCGCTCGCGCACGGACGAC<br>GCCGCCGCCGAGCCGCTGCAGCCGCTGCAGTCCGACCTCAAGTCGCCCAACGGCGTG<br>CCGCCCGCCGCCGCCGGCTGCTAGCGCGCCCGCCCGCTGCCGCAGCCGCCGCGCCCG<br>CCGCGCAGCCCGCCCGCCGTGCGCTAGCGGCCTCTGGTGGCTAGTGGTGACCGACAT<br>GAGCTGCTGATACACCAGCGGCTGCTAGCGGCTGCTGGCGGCTGCCGCGCGCTGGTC<br>GCGGCGCACACATCTACCTCAGGTCGGCCGCCGAGCCGCCGACTCTTCGAATCGTAG<br>CCGTAGCCTAGTGTAAATATCGTAATGATCGTCCGACTCGCGGGATCGTCTCTGACTGT<br>AGTGTATCGGGCTCCCCGGACCCCTCCGCATTTATTACATTAGCGCGCCGGCGCCGCCT<br>ACAAGCTTGCAGACGCGAGGCGGCCTCGAGGCGTCGCGTCGCTGTCCGCTGCAGACG

	CGAGGCGGCCTCGGGAGCGACGCGTCGCTGTCCGCTGCAGACGCGAGGCGGCCTCG<br>AGAGCGACGCGTCGCTGTCCGCTGCAGACGCGAGGCGGCCTCGAGAGCGACGCGTC<br>GCTGTCCGCTGCAGACGCGAGGCGGCCTCGAGAGCGACGCGTCGCTGTCCGCTGCAG<br>ACGCGAGGCGGCCTCGAGAGCGACGCGTCGCTGTCCGCTGCAGACGCGAGGCGGCC<br>TCGAGAGCGACGCGTCGCTGTCCGTTGCAGACGCGAGGCGGCCGCGAGAGCGTCGG<br>CTTTATTGTTTTATGAGAGCTCGACCGGAGATGGCGATGCTTTTCTTTTCGTCTGACGAT<br>TCGTTTTCATATCAGGAAAGTAATATTTTTGCTGAGACGATATTGAGTGAGTCGATTTGT<br>CTCGACGTGAATCCTATATAGTTTACCTCGACGTTATTATATAGTTATAGATCGGTGTCAT<br>TTTTGACATAGCCTATTAATGTTAAGTTTACCGTTTAAGATGTAAAGCGTGTATAGTTTT<br>ATGTAGGCGAGATCGTGTACAAATTATTTTTATTGATTTCTTGACGTATAGTTCGGTACC<br>TTGCTACTGTTTCAAAGGTAAAAGTAATTTTGTAACGGGTGTCAAATTCGTTGAAGTTG<br>TTATGTTGTTGTGGCGAGTACTGTTATTGCAAACGTACTTGCGATATGAATTTGTCACTT<br>GTCCTAATCGATGCAGAATGCAACCGCTTATAATACTCAACTGTTGGATATACCGCTTT<br>ATATATCAAGTTATTTTTGCTACTTGTATAAAAAATAATAATGTCAATATAAAAATTTAA<br>ATTAAATTAACCACCAAAGATTTGGAAAATAAACGTCTAAATTTACAATTCTTTTTATG<br>CTGTTACTTATTGTAGCTTTGTTATCAATGTTTGCAAAGATAATTATGTAATTGTATTAGA<br>GTAATTATACAGGGTCGTTTCAAATTAGTTGTAACTGGTTTTAGTAGGAACGTGAAGCA<br>ACACCCACAGTTTAGTCATAATGCGTAAGTTAAACGAGAAAAGACGAAGAACGTGTT<br>ATTTAGTGTTGCCGGCCATACGTAGCGATATTTATATTGCACAAACTACAAGCAGGATG<br>CGTGTTCTCATGTAGTTTGTGCAAGCGAGTACTTACTAGTACCAGTTAAGTGACAACGT<br>TCAATTTAAATGACTTCATATACCGCAACAATTATTATTTAACAACTTAATATTATTGTGT<br>ATGAATGATTATAGATGTATGTCATAATCGTGTTGCAGTTATCTCATTAAGAATTTATGCT<br>GAAAACAATGTGTTCCCCATCGACTAAATGCAATAGATAATACCAGCCACTATTTTAA<br>ATAAGTAGTTATATTACTTACATACTATTATTTTAAACAAATTTACATGTAGGTACTGAA<br>AAGCCAAAGTATATGTAAAGATCGGCGCACACCGGCGAACACACGATACAGGATTTT<br>AAGTTTTATAACCGAAGCGGGAACAGATCGCAGCGGAGCCACTGTGTGCCCCCAGCT<br>CAAGTGCTGGCCCTTATGTGCAAACCTAATCAGTGAACTATGTGCGGCTATAGCTACTA<br>TGTATGGTAATATCTCACTAAGAAATTAACTAAGTATGTTACTGAAATAGTTAATTTATG<br>TAATACGAATATATTTATGAATGTAAGATAACGAGACGTAAAGTTAAATGAGAACATAT<br>ACATAAATATTATGTATTCTTAGAGTTCAACATTTGTTGTGTTCATATTTTTCCATTCATAT<br>AATTACAGTTTTTATAGTAATTTAGTTCACTGAAATAGAACTGTAGTAACACAATGTAAT<br>AATAGGATATATTATTATAACTATGTAGTTCCTTTTTATTTTCTTCACATTAAAATGTGCA<br>GTGATTTTTGGCAGCAAGTATGTAGATATTATTGACTTTGTTAATTTAATTAATGACGTA<br>ACGCGAATTAGACATGGTCGTGGGGTGAATTTATTATGGTTTCACTATATGAATTTTCGA<br>TATTGTTTTCTATTTGTAACAAAAAATATCTAGACGGCAAGATTTTAGTTAAATAAAATT<br>ACGACACTCAAGCGTGTTAGCTACATTATAGTAAATAACTGTAAAAAATATAATTATAA<br>TAAACATTCAACCAAAGTTAGACTCCAATATATACACCAGTCGTGAGTATTCGAGTCG<br>GCAGCAGTCGCGGCTGGCCCGGCGCAGTAACATAGCTAACGATAGTGTGGTATAAGG<br>GTATAACGAGTGCCTTCAGACATCCAGCCTTCTTGCATGCAGGTTTGTTGATTGTGTGA<br>GGTGGACACCATGCCGACCCGGCCTAACATACTCTCTATTATACACACCTTTGTTTTAC<br>GAGTAACGCTAGAATTTCGATAACAATACTTTCGATGGCATTTAAGTTGTTTTCATTTGA<br>ACACAATCAAGAACCTCTGCCACAGAATACCAATTCAGCTTGGTTGTCGTTGGATATG<br>GGCGATGTTTTACGAATGGCTTACCGATAGTATTTTATTTAAGTCGTTGACCACCTTTAT<br>ATTGTGAATTAGGCAGAATCAGGTAAAAGTAATGTAAGTACCTAATAGTTGAGGAGCG

	CGAGGGGGCGAAGACCTGGCCGACGGGGCTAGTACAAGTACACGTTTGTGTTGTTGT<br>CGGGGGCACAAGGCACGTTGCATCACTGATTGAACCATTTTAGTAAAATCGAAACGAT<br>CTCCGTTGAATCCGAAATCCATATATTGTTTTATATGTAAAATTTGCATAGTGCCTTTTGC<br>TCAATTATGAGGAATGTCGTAGATAGGTATCGTTAAGCGTAACCTAAATATATTAGAAC<br>TATAATGTAGTTCCATTTACGTTATTCGCTAATATTCCAGTGAGTGAGAAACACAAAGC<br>CCCACCAAACTGAAGCTCTCGGCACTGGAATGTTACGCGGCGTAACGTGCAGCGCGC<br>GTGCGCACCGGCCGAGCGACCGCGCTCACACGAGCGAGCTGAGACACTCCACGCCA<br>ACACGCTCGCACGCACCCTACACAACACTCTCATAATTACCATCTTCTATTTCCTCTGA<br>AATTAAAAAAAGAGAAAAAATGTTACATCATCTGGTGTTCCCTTTATTAGCATTTATTT<br>AATTAGAAAACAAGCCTAGAAAACGTGGAGCGAGTGATGCGTGGAGTGGCTGCGGC<br>GAAGTCTCGCACAACGTTGCCTCACACAGCGTTCCCTGCGGACTGTGCCTACTATCGA<br>CCTTTGTTACTTACGCTCTTGGTTTTATCAGTATTTGTTTAATTGTGATTATCACTTTACAA<br>GGCAGTTACATTACCTCATTTTTAGTGATTATATTATGCATAAATTATTGACTGATAAAAT<br>AATTTCCAAATGATTTATTTCCTTGTGTGAATTGTGTATGTTTTGTATAAGTGTCGGGGTA<br>GCGTTGTGCTATATGCTCAATTTAGATACAATTTCACCAAACGATTGAGATAGTGTACA<br>ATTTTATATAATTAATTTAATAAAAAACCGAGTACCTATTAATAATTTTAATATTTATATTA<br>ATAAAAAAGGTTAAAAAAA
InR2<br>TRINITY_DN6759_c0_g1	>TRINITY_DN6759_c0_g1<br>ATTTTATTTTCAGAGCCTGATTAGAGCATGAATTTTTTAGGGGCGCAGTTTTGAAAGCT<br>CGCACCGGGTGCATAAAAGGCTAGTTACGGCACTGCCACCAGTGATGTAATCTGATGA<br>CGAGTTGATACTTACCGCATTCGGATAGACTCGGAAACACGATCGTCTTTTATCTGAAT<br>CGATTCAATTACCATCGATTACCAAATTAAATTAAATACCTAATTACCTAACTACTACAA<br>TCAACAAACTTCATTTATACTGCTGTCCTTATCCCACACTGTAATTGGCGTAAAGACGG<br>AAAGAGATAACAATATGTCACTATTCGTTTACTTGTTTCCAATGGATTTCTTTGTTGTTA<br>ATACTTTAGAAATTGATCCAGACAATAGTAAAAATCCCATGATCAATATAGGGCCATGG<br>GAAACGCTACACAATGTGTTTTCCCATAAATGTAAGGTACTTACACTGTAGGCTATTCA<br>GTCGTTTCGTATTGAGGAAGCGGTTAGCTGCGCGCACGCAGAAAAAGTATAAAAAGT<br>TCGGCAAACTCAAGGTGTCATGGCGAAAACGCTGATATGGTTGCTGGTCCACTCCCTC<br>CTTTTAGGCGGGATAACACCCCAAAGGTTGGATGTATCGAGCACAAACAGTTCGAGG<br>ATGTGTGAAGGAAGACTCATCAGGAGCATCATAGAGCTGAAGAAGCTAGACGGATGC<br>GTCGTCATCTTAGGAAGCCTGGAAATATTTATAGAGAAAGCGAAGAAGGAGAATTTTG<br>TCAATATGACGTTTTGGGACCTGAGAGAGATAACAGAATATCTAGTAATTTATCGAGTG<br>AGGGAATTGGAGTCTGTTGGCCAGTTGTTCCCGAATCTGACGAGGATAAGAGGACTG<br>AAGCTGTTGAACAACTTCGCCTTGGTTGTGTATGACAACACGCACCTCAGAGAGATTG<br>GTCTCTACAGCCTCCTCAAAATCGACAGAGGAGGGGTGTCAATGTGGCAGCTGCCTC<br>AGGCGTGCTTCGTGGATACCATTGACTGGAAGGTGCTGGCTCCTAAGGCCCGGCATGT<br>GATCAGTCCACCAGATATCCAGGTGCAATGCAGCATACCGTGCTCTTGTTCTAGGAAT<br>GCTTCGTTGAATCGCTGTTGGAATAATAGAAAATGCCAGTTATTTCTGGATGGACCAGA<br>AGCTGACAAATGCAATGAGCAATGTATTGGATGCCGGAAGACTAATAAGAATATTTGC<br>TTTGTCTGTCGTCATTTCACGCATAAGGGAAAATGCGTGACTCAATGTCCACCTGATAC<br>AATCCTTCTTCCTGACAACCAGTATTGCGTCACCGAGGAAGAATGCTGGGACTTGGAT<br>CGATGGGTCTTCAACAACACCTGCGTTGCTGACTGTCCGATAGACTATAAAGTGAACA<br>ATAAGACCCATCCCATCTTCTGTGAGCCTTGCACTCATTGTGATATTACATGCGGTGATC<br>TAAAAATACAGTCTCTGGGCTCGATTCAAAATGCAGAAAGATGTGTTTACGTGAACGG

	ATCGTTGGTCATACACGTCAGAGCACTTGCAGGAGCCATGAGCGACCTTAGAAAATAC<br>CTCGGTCACATCCAAGAAGTTTCACAATACATAGCCGTTGAAGGCACCGATATCATAT<br>CTCTAGATTTTCTCTCATCACTAAGAATAATCAAAGGCGAGAAGTTAAAAGATGATAA<br>ATATATTCTCGATGTTAATCACAATAGCAATCTTCAAACATTATTTACTCCAGCTGTTAC<br>AAAGAATCTCATAATCAAAAATGGTACAGCACGGTTTGATCAGAACCCAGTACTATGC<br>ATGTCCAGAATAGATGAAGTGAAAAACCGGCTCCCGATGAAGCCAACATTCTTGGAG<br>GTTCCTATAGGCTCCAATGGGTACAGCGGTGCATGTAAAGAGGTGTCTTTCGACTTCA<br>AAATAGCAGAGTCCAATGAATCTTCAGTGTTGATAAAGTTTTCTCCTGTTCATGATCAC<br>AAAATACATTACTCTGCTTTATATGTGCGATTACCACCAGGAGTTCACACGGCGGTAGT<br>TCCTGAAACTTGCAGTGAGTTCGAATGGCACGCTACTGATGTTCCTGCCGAGTTCGAT<br>GAAGGTTTCGGTCTAACGAAATTGAATTCATTACAACCAGCTTCAACTTATGCTCTCTG<br>TATCGAAATATACGATCCTATAAAAAAACTATTGATCAGGAGCAATATATTCAACTTCT<br>CAACGCCTGTAGGAATTCCTGAGCCACCATTTATCGTAGAGTTAGTTGCGTCTGCATCA<br>GATGTTGTTGTCATACGATGGGTCGATCACAAAGATTATATGCCGCACATCGATTACTA<br>CGAATTGGATGTGGCTTTGATTGAAAGGAGTACAACGAAATCTGCGGTAGATTATTGT<br>AAACTTAAAGAAGATGCATATGAGTTAGACTACACTCGTCATGCATTAGTAAGGAGAC<br>CGCCTCCAGAATATAACAGAGGCTGTGAATCAATGTGTGGCGTCCTAGCTACTGTTACT<br>CCTGGAGCGATGGTAGAGGAGTACTTTGACGTCTGCGATAAAATACATATCGGTTGTG<br>GTAATCAAGAAAGCATGATATTAAATAATTCAACTTTTGGCAACTACGAGTATGTACGA<br>ACTCTTATTTTGTTAAATATTAGCAGCCCCAGAAATGATTTCCAAGTCGGTGGGCTGGC<br>ACCCTTTAGTGACTATAAGTTTAGGTTAAGAGCTTGTGCAGGTGGTCATTGCAGCAGAT<br>CATCAAAAGGTGTGGTCAGAACATTCAGTTTAAAAAATGCTGACATACCTTCTATCAC<br>GTACTTGTATGCGAATCAATTCGGGCATATTTATGTGAAGTGGAGTCCTCCAAATGTAA<br>CTAATGGCCCAATATTATCTTATTCCGTCGAAGTGTTGCCTAGAATTAATATCAATAATC<br>TGATGCCTCAATCTTGGTGCATTTCAGCTGATGAAACATGGATGTTTATTCAATCTGTG<br>AGAGTGCCCAAATATTTAGTTCGAGTGTGTGTCAAGACGCTCGCTTCAAGTAAATCTT<br>GTGGTGATTGGATAAAAATTACAAGTGTTACTGAGTCCAAGACGATTTTGTTGTGGGCT<br>GGAGTGACTTGTGGCTTTTTGATATACATTGCGTCGTGCATAGTGGGTTGGTTTAAGAG<br>GAGGTCTCGCTATCGCGTCGATGTAGTGCCATTAGTCGATAGTTCATCTTCGTCACGTG<br>TCGAGAGCGAGCCCCCGGCGCTGATGCTATCAGATTTCGTGCCATTTCATACGATATCC<br>TTAGATTAATGTTGTATTGTGATACTACTGTAACGATTGATAAAAAGTGTATTATGATAG<br>TTGACATCATTGCGAGGAAAAGCCTTTTGTTAACGAGTTTTGGCTGACTGTAATACATA<br>CCAGTACTTTGTGTACTGTGATGAAATCCTGACGTTTTTCGGGACGTGTGTCCGCTTAA<br>ACGTCCATTTTTGTGATGATGGTTTTAATGAAATTGGTTTTATGTTACGCAAACACTCTC<br>GTCTGTCATTTTAACAAAGCCTTTGAAATGTTTGATTTTAAAAGCTTTGCCTGATAATTT<br>CCTTACTTTTACGCAGTGCAATTTCAAAATGTATATTTAAAAATAACATTTTGTATGGAA<br>GTTGTTATTATTAAAAATAAATGAGATTAACAATGTGTCAG
PDK,<br>TRINITY_D<br>N468_c0_g1	>TRINITY_DN468_c0_g1<br>GCGTCAGGTCGCTTCATTGCATTCCAGTAATCCAGTAGAAGTTGTCAGTCCGCAACGC<br>GCCGTTTTACCGAACGGACGTACGCGCGCAAATAACACTAGAAAATTATCGTTGTTTA<br>TTTATTTATCTTGATTGCATATCTCTTAAATTTATTTGTGTTTAATAATCGTCTGAACTCTA<br>TTTGTGAAAAATAATTCTTGATTTAGAACAATAATCAACCTTCATGAACTGTGATCGTG<br>TTTTTGACCGTTTCAAACAATAACAAAACGGTAGAAAACTAAAGTGCGAGACAAGTC<br>GAAATGAGCGGATTGACCAACAGAGTTAAAAGAGGTTCGAGGGGTAGTGCAACTCTT

	CTCGAAGCAGCTAACAGGATTTTGCGGTTACTTGGCGTTAGCTCCACGAAGCGCGGGA<br>AACAGCCCTCGCCCAAATCTAAGATCAACGGTCGAGCGTCTAGTGAGTTACGGGCCC<br>CAATTGCTGCGGCCGAGACCATAGTGGAGTCGCCGATCAAGATGCAAGCAGCGCCTG<br>CGCCCGCACCGGCCGCGCCGGCGCCCGCGGCCCCCGCGCCGCCCGCGCCCGCCGCG<br>CCCACCGCCTCGCCGGCCGCCAGCCAGGCACAGAAGCCCACCAAGCGAACTGCTAA<br>AGACTATATTTTTGGCAAATTAATTGGCGAGGGATGTTACAGCACCGTATTTCTTGCAA<br>AGGATATTCACAGTGGAAAGGAATATGCAATTAAAGTGTGTGAAAAACTTCACATCAT<br>CCGAAAAAAGAAAAGAGAGTACATTAAACGCGAAAAAGATGCCTTGAACATGTTGTT<br>CAATGTAGCTCATGGTTTCGTGAAACTGTACTGCACATTTCAAGATGAAGAAAGACTG<br>TACTTTGTCTTGTCCTTTGCAAAAAATGGCGAATTGCTGTCTTACATAAATCAAGTTGG<br>CTCTTTTGAGTTAAATGTAGCTAAATTTTATGCGGCTGAATTATTGATGGCCTTAGAGAA<br>AATGCACGACAAGGGAATCATTCACCGTGATTTAAAACCGGAAAATATATTATTAGAT<br>GAAAATATGCATTTGCAAATTGCTGACTTCGGAACGGCTAAAATTCTTGAACGTGAAG<br>AGATTCGTGCCTCACCGAATAATGCAGATAGTGACACGCAAAATGAACGCTCTAGAA<br>AAATTAGTTTCGTTGGAACACCGCAGTATGTTAGTCCAGAATTATTACACGACTGCGTA<br>GACACACGTGCCTCAGACCTGTGGGCGTTGGGATGCATCATTTACCAAATGATTTCGG<br>GCTTGCCTCCGTTCCGCGCAGCCACAGAGTTTCTCACCTTCCAAAAAATCCTCAAGAT<br>GGATTTCGAATTCCCAGAAGGGTTTCCCGCTGACGCAAAAGATCTTGTGGAAAAGCTC<br>TTAGTTCTAGACCATACCAAACGACTTGGAGCTAATGATAAAGGAGAAACATATGATA<br>GCATTCGCAATCATCCGTTCTTCGCTGGAATCGACTGGGACAGTTTGTGGGAGCAGAC<br>GCCCCCTACTATCAGCCCATACTTACCCGGAGGATCTTTCGGATACAATGTACCCGATC<br>ATCTTGAACCAGGACTGGGCAACAAGCAACTCGTTCGACTGTGGGAATTCGACTTATC<br>TACCTCTAAAGGAATCTTAAACATCAGCCCCGAAGAAAAACGTCGTCGTCTCGAAGT<br>GCAATCTCGGGAGAGCAAGTGGCACCAGTTCGTGGATGGAGAGCTCATTCTGAAGCA<br>AGGCCTGGTCGACAAGAGGAAGGGACTGTTCCCGCGACGCCGCATGTTGCTGCTCAC<br>CACCGGACCGCGCCTATTCTACGTCGATCCAGCTAACATGGTGCTCAAGGGAGAGATC<br>CCTTGGTCACCCGAGTTGCGGGTCGAAGCCAAAAACTTCAGAATATTTTTAGTGCACA<br>CGCCAAACCGTACATATTATTTGGAAGACCCCGATTCGTATGCTTTGGAGTGGGCAAG<br>CGTAATCGACGAAGTGCGCATCGGTACATATGGGCGGGACACGACTTAAGCGACGGC<br>GCGGCCGCAGCTCCGAGTCGCGCCCGCGCCGCCGCCGCGGCCCGCGGGCCCAGCGC<br>CCGCTCCTCGTACCTCACACGGCGAGTTCACACTTCTAGGTTACTTCTAGCCTTGATTT<br>AGAGCATGGCAAATTCCAAGCCGAAAGCTTTCGCGTCGATGATCGACCTCGTGCGTGT<br>CCCTTGTCCCGCGCCGGCCGGTGCGGGCCGGCGGGCGTTGCGGGCGCCGAATGCGGG<br>CGCGCTACATATGTTCACAGCGCTCGCACTCGCCCGCGCGGCCGCCCGCACGCCCGC<br>ACGCGCCGCCGCGCCGGCCGCTGACGAGACGACGACGATCGACCCTGCTCGATCACT<br>GTGGATACTGAATGACTGATTCCTATTAATAATAATATCCCGACTATCTTAAATTATTCTA<br>ATTTAAGTTCCTTATTTAATTGTGGAATACCGACGATGATGATGTGTAAATATGTCTTAA<br>ATTATTATTGCGATTGCTTAGTAATAGTTAATTGTGGGGCGATGTATGTTTTATTGTATAC<br>AGGCCTCTTGTAAGAATGGACTTGTTATTCACCACCTGCGTGTTCACGAAACCCATTCC<br>GGTTGGTAACTAGGCAATTTATAGCTAAATTTAATATTTATTTTATGTTTAATTATTTGCC<br>AACGGAATGTGACTGCTGTACAGTTATAACCGTAGAAAATTATGATTGGTATGTTTCTA<br>TATTCTACTTCAACTATGTTTATCGGTGACAATTGGAAAGTAAAGATAAGAATTAGCAA<br>AGTTGTATATTTTAAATTGAATTAGCAATTGTCACACACTGGTTGCTGTAGAAATAGAA<br>GCAGCTAGACGTAAGGAGGGTAGCTTCTTCACTAAATAATTGAGAGTTGGTGCGAACC

	GATCCGATGGCGATCGGATGCCTTACTTTCACGACTCAGTTGAGTATAGTCTGCTCTGA<br>GATAGGAAATGAACACATTGTTGAGGAGTTTCAATGCTTTTTATTGTGCAAGTTACTTG<br>TGCCCTTTAATTCTAACATAGCAAAGGTATACGGATAACGCCACCAAAGGTGACAATT<br>ACTGCTATTTCATTGCTTGGTAATTAATTGTCTAAAATATATTATTTTTGTTAATGCAGTC<br>GAATTAAGACATTGGGATTGCTTAATGGATAGTGACTAGTGCAAATTTGTTTTTTAATGT<br>TTTGTGTTGTTATGAACAGTGATTTTATGGCCATAGCAAATATTCAATGTCAATGTTCTA<br>AAATTGTATTTAAATTGTAATGGTGAACTTATCATTTAATCATTTTATAATAGCAGGTATA<br>TTAAAATGCCTAAAACTTGCCTGTAATATTTAACATTAGGGCTTAATGGGATTTAATCCA<br>TAGTTCAATACGAAACCCTCATATAGCAAAATGTAGAAGACTAAGAATTCACTAAGTA<br>GTATGAAGGACTGTTTTATTCATTTCATAGCGTATTATGGGCAATGAGATACAATATTAT<br>TAATTGGTCAGCGTAAATTGGACTTTTCCATGACATTGTTATGAATAATTAAAATTAAA<br>ACCTGCATTTAAAATTTAATAACTGTTAACTATTGTAATTATATTTACAACATTTGTATT<br>GCATAAATTCCATAAAGTAACAAAATAAGAAATTGAAAATAAATTGAAGTTGATTTTT<br>AAA
TO1,<br>TRINITY_D<br>N1529_c1_g<br>1	>TRINITY_DN1529_c1_g1<br>ACACTGCCCTCATTCATCACGCCGTGCTCCCGCTCAGATCCCCAGCTGAACGCCTGCA<br>TAGAGAAGGTGATCACCGCCGCCGGCTCCAAGTTCACCGAGGGCATCCCAGAGCTGG<br>GCATCAAGCCGCTGGACCCCGTGGAACTGGGCACGGTGTTCGTGGACAACCCCGCCT<br>TGAAGCTCACCTTTACTGACACCGTCGTTACTGGACTGAAGGGATTTAGGGTCAATTC<br>ATACAAGATCAACCCAGACAAGGGTAAGGCCACCCTGGACTTCACCGCCAACGTGAC<br>GCTGAAGGCGCACTACGACATGGACGGACAGGTTCTCATCCTGCCCATCAGGGGCAA<br>CGGACAGTCCAGGATCAAGATCACGAACCTAAACATTGTGGTGAAGTACGACTTCGT<br>AGAGAAGGACGGGCACTGGGTGGTGCCCAGCTACAAGGACCACTATAAGATGGACC<br>GCGCGCAGTTCAAGTTCACGAACCTGTTCAACGGCAACAAGGAACTCGCACAAACG<br>ACAGAACGGTTCACTAACGAGAACTGGGAGATCATTATGAGCGAGATAGCTCCCGGC<br>GCGATCAAACAGATCATCAAGAAGTGCGTCGATCAAGTCAAGAAGTTGTTCACATCT<br>GTACCGGCCAGAGTATTGCTACCTTGAAGTCCTGAACGGTGAAACCATCCACCAAGTA<br>ATAAACATAAACTGGGAGCCTGTGATGCGGGACATAGGGCCGGTCGCCTTCCAACAA<br>ATCATCAAAGCGTGCGTCGACGAGTCCAAGAAGCTGTTCGCAGCTGTTCCACTCAGC<br>CAGCTGCTGCTGCCTTGAGTCTCTTCTCCGTTTGTGCAGTTACATCTCGAATGTGCAGG<br>TCTTTTTTGTGGAAGCCAACCTTTTTCGTACTGAATATAGCACTCTTCTTCTGTTGTTATT<br>ACCGCCGAAGGAACTTTTCCTACAAATTGGTAATGATTGTTTCTTAGATAAATCACATT<br>GTCTTCGTTAGTAGGATTGGTGTTCAAAATTCAGTAAATTATTAAAGTCGGTACAATCG<br>CTAAAGTCTGCGCTCCTAGATACACCATTCTACAATTCTACCTATCTGAAATCCATCTAT<br>CTGTCGAAGATCAATAATTTTCACTACGTTACATTAGGACATTCAAAAAGGATCGCAC<br>AACTCGAGGAGTAACTGCATAAAGGAACCTTTAATTTAATTTTTCAACTATTTTGTTCG<br>TTGAAGCACGTCCCGCGTAAATCTGCACAAAATAACTTCTGTACAAGACTGTGTTTGA<br>TCATTTGTTTGTAGGCGCATAAGATAAGTTGGTTTGTTAGTAATTGTGATAATTAGCCTA<br>AGTTAGTTAAGTTGGTTTTAGATTTAATTTATTCAAAAATAAATTGGTGAAAACGTGAA<br>AAAAAAAAGAAAACAAAA
TO2<br>TRINITY_D<br>N3829_c0_g<br>1	>TRINITY_DN3829_c0_g1<br>CAACAATTATGAGACGAAATTGAGAAGCAATCATATTTCGCAAGCGTTCCCGCCAATT<br>CGGATAAATATGGCGGGTGAACCGCGCGCGCCTCAGTCGCCAAGGCGCATGCGCGAG<br>AACTGTCACTTTCGTTCGAAAATTGCGAATGTGTTGCAGAGAGTGAAAAATAAAAAA

	GAAACAAAAATGTTTGATTCATTATTGCTTGTGGCTTTTTTGACGAGTTTCGTGAATGCT<br>GAGAATAGTTTACCTGAAGATTTCACGAGATGCAGACAAAAAGACGCAAAACTCAAC<br>GAATGTCTCAAAATGGCTGTTCCTGACGCACTCAGAAGGATGAAGAAAGGTATCCCC<br>TCGCTGTCAGTCCCGGAGATGGAGCCGCTGCACGTGGACAGCATCAACGTGGACTCG<br>GGCTCCGGCCCGGTCGTCATCACGCAGATGTACAAGAAGATCAAGCTACATGGGCTC<br>ACGGATTCTGTGCTCACGCTTTACAAAGCTGATCTCAAAAACTATAGACTCAGGACAG<br>ACTCGATCACGCCAAAGATGGAGTTCATCGCTGACTACATCATGAGGGGCAGGATTCT<br>TCTGCTGCCCATCCAAGGAAAGGGTATCGCTAACATCACCATGGTGAACCTGGTAGTG<br>AAACACGACCTGATCGGCGAGCCGGTCGTCCGGGACGGACAGACGTACATGCACATC<br>AAGGACTACAGGGTCAAGTTCATCCCGCAGCGCGTGCTGCTGCACTTCACCAACCTCT<br>TCAACGGAGACAAGGTGTTGGGAGATAACATGAATGAATTCCTGAACTCAAATTCGG<br>ACCTCGTCTTCAATGAACTGAAAGAATCCTACGAGAAAGCCCTGAGTTCAGTGTTCCA<br>GAATGTCACCAACGAAATATTTGACAGAGTGCCAATGAACAAAATATTCCTCGAAAA<br>CTAGTTTGTCTCCATCAAGCTATGTAGGCATAGCAAGACTTTTAGCCTAGTTGCAAAAT<br>AGATGTAAGACAATCCTTTTGGGTAAGCGAGGAAAAGAAATACACCTTCCAGTATGTG<br>GTATTTATTAAAGCTGCTTTAATTAGACTTCGTTATATTAAGTTACAATAAATTTTATAAT<br>AATTTAGTTTATAAGTAAAAAAAAGTACTTATGAAAATTTGATGTTGTTTTATTTGGTTT<br>TACGAATTTTTTAAGATTTGCCAGCTGAAATTTTGTGGGAGTCGATTGAGACCGCTCCA<br>CCGCGACTGGGAGGATCGCAGACTCTTGCGCACAGGAACAACTAGAACATTGAAAA<br>AAGCGCCATCTCTTATTCGTTTCTAGTAAGCTAGTCGATAGGTAGGCTGGTGCATGATG<br>GGCTCCGTGTCCTTCTTGGGAGGTTTCCTCTCGTTCAGCTCCACGTCGGGTCCCTGGTG<br>GTTCAGACGAGCTTGCTGCGCGTTTCGTAAGCAAGCTGTTTTGCAGAAAGCCTCGTTT<br>ATTATGACTAATATTCCAATCAGGAGTAAGTATATAATGAGCCCCAATATGAGGGCATC<br>GTACTCCATGGTTGAAGATATTAGCAAGCCCTGCGAGGCCTGGTGGCTCTGATGAGTT<br>CCCTGATGGAGCAGAAACAAGCGAATATGATTACTAGAAAAATAGCAATCTGCCAGA<br>AATTTGCCACCAGAGATTCGCTTACTGGCCCCGCACTCTTCGCCATCAGTTAGAATAA<br>ACAAGCCAACGGGTTCCCATTCGCATTCCTAGTCAGGCGGAAGAGTTCTCTGATGGAA<br>AGGAATGCAGCAAACACGATGACCAGGAAGAAAGAGGTGTTAAATGCTTGTGTGAG<br>CACAGAAACCACGCTCATCATCTGGATGAGCTCAGGGTGTCTTCAGGGGACCATCAC<br>GGTGAATTTCGCTGGAGTGTTGGACGGATTTGACATGGAACTGACCACAGGAACTGA<br>CAATCTGTTCTCTGTTGACGGACG
TO3<br>TRINITY_D<br>N9355_c2_g<br>1	>TRINITY_DN9355_c2_g1<br>CGATGTTCGTCTGTTCGCACTTTTAGGGCATGACCAACTGTAGGAAGCGAACACTATT<br>AGCCAAGACGTGTCCTCTTTGTTCAAGCACTGCTCACATGACATAACCGTAATTACTC<br>ATCAACTTGCTCGCACACCATAAAGAACCTTACAAATCCTATAATTTCTTCGTCCTGGC<br>GGACCGAGCCAGTAAATAGTTCGCAGGCGGTGGGGAGCCCACTCAGGGATGTCGAA<br>AATCTTGAGCGAATAAAACACATAGCTCCTTGCTTTCACCTCAAACAAAATGAGCCGT<br>GCGCCCGTCCGCCTACCATCCAAGCGCGAGCCTATTGCTAACTAAAACCGCCGAACA<br>ACGGACGCGGGCTAATTTTAAAATTCGAACGTCAAAAAAAACATTTCAATAAAAAAA<br>ATACGAATTCAAATAAAAGAAAAATAAAGTGCATTTCGAAAATCGAACGTTCAGTGA<br>AAAATGGATTGTTTGAAATATTGAAAAAGTTTTAAATTTTCTTTTTTATAAAAATCATTT<br>TGTTAGTTATTAAAATTAGAATTTTAATTTTTAACAAAAATGAAAATGGCCGATCGTTGT<br>GTTTTGTCGCTGTTGATGTTGAGTTTGACGGTTTTATGCCGCAACGGGGAAGCTGCAGA<br>AGATGCGAGTGCAGCCAAAATACCGGAATACATCCAGCCGTGTAGTCAAAAAGATGC

	AGAGTTGGATACTTGTATCAAGAACTCGTTCAACCACCTGCGGCCGTACCTGTCCAGA<br>GGTATCCCTGAACTGGGGGTGCCTCCTGTGGAGCCGCTCTTCATCGATCGGCTGGTCAT<br>GGCCAACGACGCTGGACCCGTCAGGGTGACCGCAGCCTTCAGCAACATCACTGTTAT<br>CGGACCTAGTAATTATACCATTACTAAAATCAGGTCTGACTTGAAGAAGCTTCGCATCG<br>ACATGGGGCTTGTGCTGCCTCGCATCGAGATCACTGGAAGATACGAGGTTTCCGGACA<br>GGTGCTGCTGTTCCCCGTCAGGTCTCAGGGAGACTTCTGGGCTTCTTTTGGTGACGTGG<br>TGGCCATCGCGAAGATCTTCGGCAAGGAAACCACTCGGGACAACGTGAAGTACATGT<br>TGGCGGACAGACTGCTGGTGGACTTCAAACTGAGGACCTCTCGCTTCAAAGTTAAGG<br>ATACCGTCAACCATGGCAGCATTATTGGCGAGGCGATGAACCAGTTCTTGAATAACAA<br>CGCAGCGGAGATCATCGAGGAGATGAGGCCGGCCGCCTCCGCGTCCATAGCGAAGCA<br>CTTCCAGGCGTTCGTCAACGCCGCCTTCTCCAAGATCCCCATCGACGTGTGGCTGACG<br>CCTTAGACCAGTGAGTGAACTTGTGAACAGACAGTTTTGTATGTAGTTCTGCAAGCCT<br>AGTGCGGGTTGGTTTCACGTTTTTGCGTTTATGACGTGCGGCGCTCCAATTGGCCCGCT<br>CATAACCAATCAGAGAGTTAAACGTTTCGTTACCGTGAAACAAACTCGCACTAAGGC<br>CTCCGGTATATTTACTGTTGTGAATTTTTCTAGGAGTATTTTCAACAATTTGTAGACTTTA<br>TGTTTATTAAAAGTATGATTTGATGTTAGTACAAATTCCAAATGTTGAAGATGTGTGAA<br>AAACCGAGTGTATTTTTTCTGAATTCTTTGCGTAGCTATATTGTATGCAACCATTTCTTA<br>AGGTCAATTACGACTGACAATATTTACTTGAATGACATGATATTGATAATTGCCTCAATC<br>GTTTAAAGTAAACTATCAATATTTGCATTATCGGCGTCAATATCGTCAATCAGAATTGTC<br>CTTTTCCGTAACTCTATAAATTCCCTGACTATTTTATTTTACCAACCTTCGCGTTTCATAT<br>TTGTGTGTTGCAGTGTCTTAAGATCTCAAAACATTTTCTTATTATGTTTTTTTTTTCAAAT<br>GACAACTTAAAAACATTTAACTCGATAAAATCACTGTGTAAAGTCGAAAATATGTAAA<br>ATTGTGATCTTGATTTAATTAAACTATTCTGTAATAGATCCCTTTAACGAAATGAGATAA<br>TTTTATAACATATTCGTTCGCTCCTTTTCAAAGATATAGTGTGATTATTGTGTGTTCAGTG<br>CCTAGTGCGAGTTGTTTCTACGTTAACGCGATCGACACGTTTATGATGTTCGGTATTCCG<br>ATTGGCTCGCTCCAAGCTTTTAACCAATCAGAGACCCGTACGCGGT
Gene	BLAST results
InR1 TRINITY_DN1670_c0_g1	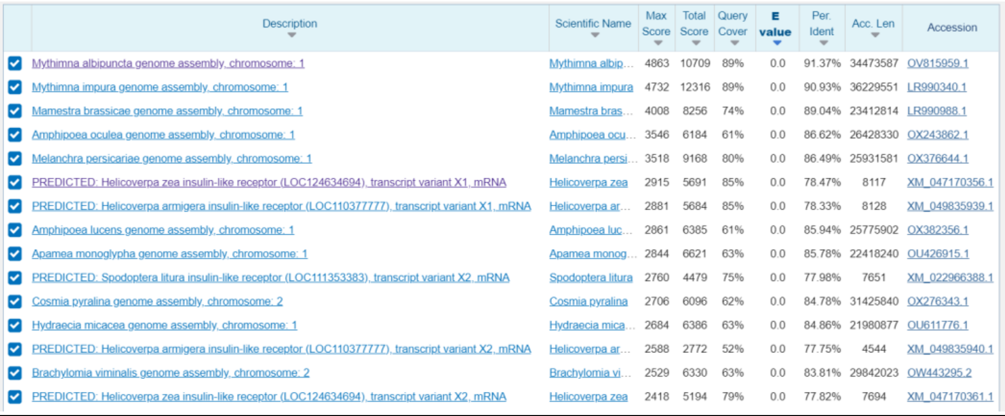

Description	Scientific Name	Max Score	Total Score	Query Cover	E value	Per. Ident	Acc. Len	Accession
Mythimna albipuncta genome assembly, chromosome: 1	Mythimna albip…	4863	10709	89%	0.0	91.37%	34473587	OV815959.1
Mythimna impura genome assembly, chromosome: 1	Mythimna impura	4732	12316	89%	0.0	90.93%	36229551	LR990340.1
Mamestra brassicae genome assembly, chromosome: 1	Mamestra bras…	4008	8256	74%	0.0	89.04%	23412814	LR990988.1
Amphipoea oculea genome assembly, chromosome: 1	Amphipoea ocu…	3546	6184	61%	0.0	86.62%	26428330	OX243862.1
Melanchra persicariae genome assembly, chromosome: 1	Melanchra persi…	3518	9168	80%	0.0	86.49%	25931581	OX376644.1
PREDICTED: Helicoverpa zea insulin-like receptor (LOC124634694), transcript variant X1, mRNA	Helicoverpa zea	2915	5691	85%	0.0	78.47%	8117	XM_047170356.1
PREDICTED: Helicoverpa armigera insulin-like receptor (LOC110377777), transcript variant X1, mRNA	Helicoverpa ar…	2881	5684	85%	0.0	78.33%	8128	XM_049835939.1
Amphipoea lucens genome assembly, chromosome: 1	Amphipoea luc…	2861	6385	61%	0.0	85.94%	25775902	OX382356.1
Apamea monoglypha genome assembly, chromosome: 1	Apamea monog…	2844	6621	63%	0.0	85.78%	22418240	OU426915.1
PREDICTED: Spodoptera litura insulin-like receptor (LOC111353383), transcript variant X2, mRNA	Spodoptera litura	2760	4479	75%	0.0	77.98%	7651	XM_022966388.1
Cosmia pyralina genome assembly, chromosome: 2	Cosmia pyralina	2706	6096	62%	0.0	84.78%	31425840	OX276343.1
Hydraecia micacea genome assembly, chromosome: 1	Hydraecia mica…	2684	6386	63%	0.0	84.86%	21980877	OU611776.1
PREDICTED: Helicoverpa armigera insulin-like receptor (LOC110377777), transcript variant X2, mRNA	Helicoverpa ar…	2588	2772	52%	0.0	77.75%	4544	XM_049835940.1
Brachylomia viminalis genome assembly, chromosome: 2	Brachylomia vi…	2529	6330	63%	0.0	83.81%	29842023	OW443295.2
PREDICTED: Helicoverpa zea insulin-like receptor (LOC124634694), transcript variant X2, mRNA	Helicoverpa zea	2418	5194	79%	0.0	77.82%	7694	XM_047170361.1

	Description	Scientific Name	Max Score	Total Score	Query Cover	E value	Per. Ident	Acc. Len	Accession
InR2 TRINITY_DN6759_c0_g1	PREDICTED: Helicoverpa armigera insulin-like receptor (LOC110369826), transcript variant X3, mRNA	Helicoverpa ar...	1886	1886	80%	0.0	78.28%	3542	XM_049844095.1
	Dryobotodes eremita genome assembly, chromosome: 17	Dryobotodes er...	1884	2378	61%	0.0	84.86%	22994139	OU823258.1
	Agrochola circellaris genome assembly, chromosome: 14	Agrochola circel...	1868	2564	66%	0.0	84.69%	19686539	OU611853.1
	Dryobota labecula genome assembly, chromosome: 14	Dryobota labecula	1858	2553	67%	0.0	84.81%	26437037	OX383297.1
	Noctua fimbriata genome assembly, chromosome: 13	Noctua fimbriata	1857	2683	74%	0.0	84.08%	18984432	LR990933.1
	PREDICTED: Helicoverpa armigera insulin-like receptor (LOC110369826), transcript variant X1, mRNA	Helicoverpa ar...	1851	1851	80%	0.0	78.12%	3521	XM_049844093.1
	Mesoligia furuncula genome assembly, chromosome: 16	Mesoligia furun...	1847	2559	68%	0.0	84.13%	29453729	OU744806.1
	PREDICTED: Helicoverpa armigera insulin-like receptor (LOC110369826), transcript variant X6, mRNA	Helicoverpa ar...	1845	1845	80%	0.0	78.12%	3310	XM_049844099.1
	PREDICTED: Helicoverpa armigera insulin-like receptor (LOC110369826), transcript variant X5, mRNA	Helicoverpa ar...	1845	1845	80%	0.0	78.12%	3375	XM_049844098.1
	PREDICTED: Helicoverpa armigera insulin-like receptor (LOC110369826), transcript variant X2, mRNA	Helicoverpa ar...	1845	1845	80%	0.0	78.14%	3536	XM_049844094.1
	Apamea sordens genome assembly, chromosome: 14	Apamea sordens	1844	2573	69%	0.0	84.61%	21033426	OX243999.1
	Lithophane semibrunnea genome assembly, chromosome: 12	Lithophane sem...	1840	2492	69%	0.0	83.99%	18234465	OX376198.1
	Cosmia pyralina genome assembly, chromosome: 14	Cosmia pyralina	1838	2244	61%	0.0	84.63%	26926653	OX276355.1
	Antitype chi genome assembly, chromosome: 12	Antitype chi	1836	2564	65%	0.0	84.71%	21853072	OX375819.1
	PREDICTED: Helicoverpa zea insulin-like receptor (LOC124636518), transcript variant X2, mRNA	Helicoverpa zea	1836	1836	80%	0.0	77.98%	3486	XM_047172636.1
	PREDICTED: Helicoverpa armigera insulin-like receptor (LOC110369826), transcript variant X4, mRNA	Helicoverpa ar...	1829	1829	80%	0.0	78.07%	3506	XM_049844096.1
	Griposia aprilina genome assembly, chromosome: 14	Griposia aprilina	1827	2496	65%	0.0	84.56%	23769850	OU744297.1
	Hydraecia micacea genome assembly, chromosome: 14	Hydraecia mica...	1827	2505	66%	0.0	84.57%	18572387	OU611789.1
	Amphipoea oculea genome assembly, chromosome: 13	Amphipoea ocul...	1820	2516	67%	0.0	84.24%	23095043	OX243874.1
	Fissipunctia ypsillon genome assembly, chromosome: 10	Fissipunctia yps...	1816	2466	64%	0.0	84.36%	25300366	OX387653.1
	PREDICTED: Helicoverpa zea insulin-like receptor (LOC124636518), transcript variant X1, mRNA	Helicoverpa zea	1816	1816	80%	0.0	77.93%	3470	XM_047172635.1
PDK, TRINITY_DN468_c0_g1	Description	Scientific Name	Max Score	Total Score	Query Cover	E value	Per. Ident	Acc. Len	Accession
	PREDICTED: Trichoplusia ni 3-phosphoinositide-dependent protein kinase 1 (LOC113496147), transcript...	Trichoplusia ni	3766	3766	99%	0.0	85.47%	3668	XM_026875285.1
	PREDICTED: Trichoplusia ni 3-phosphoinositide-dependent protein kinase 1 (LOC113496147), transcript...	Trichoplusia ni	3323	3323	88%	0.0	85.42%	3714	XM_026875277.1
	PREDICTED: Trichoplusia ni 3-phosphoinositide-dependent protein kinase 1 (LOC113496147), transcript...	Trichoplusia ni	3319	3319	88%	0.0	85.42%	3351	XM_026875294.1
	Mythimna ferrago genome assembly, chromosome: 20	Mythimna ferrago	2940	5990	99%	0.0	96.38%	26299396	OU342693.1
	Mythimna albipuncta genome assembly, chromosome: 17	Mythimna albip...	2918	5991	99%	0.0	96.16%	22725865	OV815975.1
	PREDICTED: Helicoverpa armigera 3-phosphoinositide-dependent protein kinase 1 (LOC110370086), tr...	Helicoverpa ar...	2782	4515	98%	0.0	89.63%	3738	XM_021325812.2
	PREDICTED: Helicoverpa zea 3-phosphoinositide-dependent protein kinase 1 (LOC124635413), transcri...	Helicoverpa zea	2771	4498	98%	0.0	89.55%	3657	XM_047171295.1
	Mythimna impura genome assembly, chromosome: 18	Mythimna impura	2754	5815	99%	0.0	94.82%	29725006	LR990357.1
	Mamestra brassicae genome assembly, chromosome: 17	Mamestra brass...	2608	5323	99%	0.0	93.10%	18759184	LR991004.1
	Agrochola macilenta genome assembly, chromosome: 15	Agrochola macil...	2542	5237	99%	0.0	92.58%	22486168	OU753567.1
	Agrochola circellaris genome assembly, chromosome: 16	Agrochola circel...	2532	5113	99%	0.0	92.47%	18935588	OU611855.1
	Lithophane semibrunnea genome assembly, chromosome: 15	Lithophane sem...	2525	5183	99%	0.0	92.29%	18015867	OX376201.1
	Eupsilia transversa genome assembly, chromosome: 14	Eupsilia transve...	2518	5170	99%	0.0	92.24%	15275328	OU611886.1
	PREDICTED: Spodoptera frugiperda 3-phosphoinositide-dependent protein kinase 1 (LOC118272397), tr...	Spodoptera frug...	2514	4166	96%	0.0	87.45%	3729	XM_035588878.2
TO1, TRINITY_DN1529_c1_g1	Description	Scientific Name	Max Score	Total Score	Query Cover	E value	Per. Ident	Acc. Len	Accession
	PREDICTED: Helicoverpa zea circadian clock-controlled protein daywake-like (LOC124634062), mRNA	Helicoverpa zea	870	1047	70%	0.0	83.64%	1896	XM_047169470.1
	PREDICTED: Helicoverpa armigera circadian clock-controlled protein daywake (LOC110377866), mRNA	Helicoverpa ar...	855	1030	70%	0.0	83.42%	1517	XM_021336905.2
	Mythimna impura genome assembly, chromosome: 16	Mythimna impura	762	1652	98%	0.0	81.28%	30280635	LR990355.1
	PREDICTED: Spodoptera litura protein takeout-like (LOC111357674), mRNA	Spodoptera litura	756	906	70%	0.0	80.37%	1457	XM_022972462.1
	PREDICTED: Spodoptera frugiperda circadian clock-controlled protein daywake (LOC118276116), mRNA	Spodoptera frug...	753	921	70%	0.0	80.44%	1511	XM_035594289.2
	PREDICTED: Trichoplusia ni protein takeout-like (LOC113505793), mRNA	Trichoplusia ni	740	869	68%	0.0	80.07%	2383	XM_026888617.1
	Helicoverpa armigera juvenile hormone binding protein brP-2095 precursor, mRNA, complete cds	Helicoverpa ar...	715	715	44%	0.0	85.10%	714	KT443974.1
	Spodoptera litura takeout (TO4) mRNA, complete cds	Spodoptera litura	575	575	46%	1e-158	79.57%	708	MF196298.1
	PREDICTED: Manduca sexta circadian clock-controlled protein daywake (LOC115441176), mRNA	Manduca sexta	552	552	57%	1e-151	75.25%	1383	XM_030165838.1
	PREDICTED: Bicyclus anynana circadian clock-controlled protein-like (LOC112044155), mRNA	Bicyclus anynana	539	539	57%	9e-148	75.28%	2702	XM_024079899.1
	PREDICTED: Ostrinia furnacalis circadian clock-controlled protein-like (LOC114365482), mRNA	Ostrinia furnacalis	518	518	50%	9e-142	76.33%	1330	XM_028322064.1
	PREDICTED: Pectinophora gossypiella circadian clock-controlled protein daywake-like (LOC126371329)...	Pectinophora g...	504	504	55%	2e-137	74.30%	963	XM_050016636.1
	PREDICTED: Leguminivora glycinivorella protein takeout-like (LOC125229526), mRNA	Leguminivora gl...	490	490	56%	4e-133	73.99%	2589	XM_048134389.1
TO2 TRINITY_DN3829_c0_g1	Description	Scientific Name	Max Score	Total Score	Query Cover	E value	Per. Ident	Acc. Len	Accession
	PREDICTED: Helicoverpa zea protein takeout-like (LOC124639484), mRNA	Helicoverpa zea	929	929	53%	0.0	82.87%	1043	XM_047176872.1
	PREDICTED: Helicoverpa armigera protein takeout (LOC110373672), mRNA	Helicoverpa armigera	909	909	53%	0.0	82.16%	1056	XM_049842922.1
	Mythimna impura genome assembly, chromosome: 15	Mythimna impura	798	1855	80%	0.0	89.94%	30436742	LR990354.1
	PREDICTED: Spodoptera frugiperda protein takeout (LOC118275183), mRNA	Spodoptera frugiperda	781	781	43%	0.0	83.14%	1270	XM_035593055.2
	Mythimna ferrago genome assembly, chromosome: 17	Mythimna ferrago	726	1882	79%	0.0	87.30%	26415199	OU342690.1
	Mythimna albipuncta genome assembly, chromosome: 20	Mythimna albipuncta	687	1851	79%	0.0	85.90%	20961862	OV815978.1
	Hecatera dysodea genome assembly, chromosome: 21	Hecatera dysodea	646	1091	56%	4e-180	84.41%	18597561	HG995308.1
	Mamestra brassicae genome assembly, chromosome: 21	Mamestra brassicae	646	1146	56%	4e-180	84.26%	17699905	LR991008.1
	Lacanobia wlatinum genome assembly, chromosome: 19	Lacanobia wlatinum	639	1321	65%	1e-177	84.09%	28467094	OX388041.1
	PREDICTED: Trichoplusia ni protein takeout-like (LOC113494604), transcript variant X1, m...	Trichoplusia ni	609	609	43%	1e-168	78.48%	1115	XM_026873027.1
TO3 TRINITY_DN9355_c2_g1	Description	Scientific Name	Max Score	Total Score	Query Cover	E value	Per. Ident	Acc. Len	Accession
	PREDICTED: Helicoverpa armigera protein takeout (LOC110379121), transcript variant X2,...	Helicoverpa armigera	1304	1304	58%	0.0	85.64%	2249	XM_021338675.2
	PREDICTED: Helicoverpa armigera protein takeout (LOC110379121), transcript variant X1,...	Helicoverpa armigera	1273	1273	58%	0.0	84.83%	2272	XM_049842037.1
	PREDICTED: Helicoverpa armigera protein takeout-like (LOC126055072), mRNA	Helicoverpa armigera	1261	1261	57%	0.0	85.39%	2169	XM_049842678.1
	PREDICTED: Spodoptera frugiperda protein takeout (LOC118263038), mRNA	Spodoptera frugiperda	1246	1246	55%	0.0	85.35%	2781	XM_035574795.2
	PREDICTED: Spodoptera litura protein takeout-like (LOC111358481), mRNA	Spodoptera litura	1213	1213	55%	0.0	85.02%	1468	XM_022973636.1
	PREDICTED: Helicoverpa zea protein takeout-like (LOC124644789), mRNA	Helicoverpa zea	1169	1169	53%	0.0	85.10%	2116	XM_047184348.1
	PREDICTED: Trichoplusia ni protein takeout-like (LOC113492894), mRNA	Trichoplusia ni	1160	1160	55%	0.0	84.20%	1342	XM_026870610.1
