# Supplementary figures and images for: Multi-Omics Reveals the Effect of Population Density on the Phenotype, Transcriptome and Metabolome of Mythimna separata
Source: Insects. 2023 Jan 10;14(1):68. doi: 10.3390/insects14010068 (PMC9861010; doi:10.3390/insects14010068)

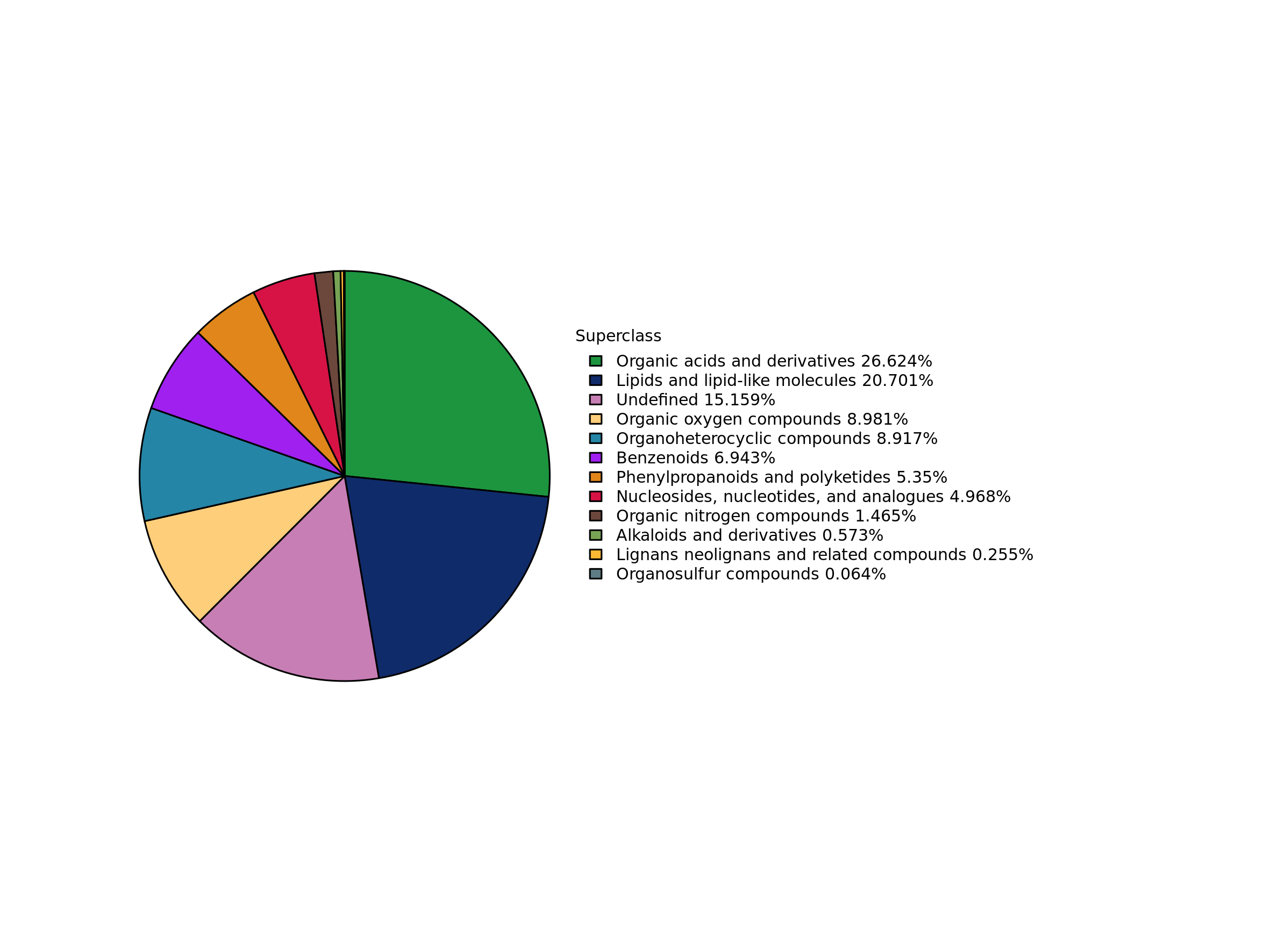

Supplement: Supplementary file 1 [file insects-14-00068-s001.zip › Figure S3.jpeg]
